# Fibroblast Upregulation of Vitamin D Receptor Represents a Self-Protective Response to Limit Fibroblast Proliferation and Activation during Pulmonary Fibrosis

**DOI:** 10.3390/antiox12081634

**Published:** 2023-08-18

**Authors:** Juan Wei, Junhui Zhan, Hui Ji, Yitong Xu, Qingfeng Xu, Xiaoyan Zhu, Yujian Liu

**Affiliations:** 1 School of Kinesiology, The Key Laboratory of Exercise and Health Sciences of Ministry of Education, Shanghai University of Sport, Shanghai 200438, China; 2011516009@sus.edu.cn (J.W.); 2121517019@sus.edu.cn (J.Z.); 2021518012@sus.edu.cn (H.J.); xuyitong@sus.edu.cn (Y.X.); 2021517018@sus.edu.cn (Q.X.); 2 School of Sports and Health, Nanjing Sport Institute, Nanjing 210014, China; 3 Department of Physiology, Navy Medical University, Shanghai 200433, China

**Keywords:** VDR, ER stress, JAK1, STAT3, fibroblast, pulmonary fibrosis

## Abstract

Dysregulation of vitamin D receptor (VDR) is implicated in chronic obstructive pulmonary disease. However, whether VDR dysregulation contributes to the development of pulmonary fibrosis remains largely unknown. Analysis of bulk and single-cell RNA profiling datasets revealed VDR upregulation in lung fibroblasts from patients with pulmonary fibrosis or fibrotic mice, which was validated in lung fibroblasts from bleomycin-exposed mice and bleomycin-treated fibroblasts. Stable VDR knockdown promoted, whereas the VDR agonist paricalcitol suppressed lung fibroblast proliferation and activation. Gene set enrichment analysis (GSEA) showed that the JAK/STAT pathway and unfolded protein response (UPR), a process related to endoplasmic reticulum (ER) stress, were enriched in lung fibroblasts of fibrotic lungs. Stable VDR knockdown stimulated, but paricalcitol suppressed ER stress and JAK1/STAT3 activation in lung fibroblasts. The STAT3 inhibitor blocked bleomycin- or stable VDR knockdown-induced ER stress. Paricalcitol inhibited the bleomycin-induced enrichment of STAT3 to the ATF6 promoter, thereby suppressing ATF6 expression in fibroblasts. Paricalcitol or intrapulmonary VDR overexpression inactivated JAK1/STAT3 and suppressed ER stress in bleomycin-treated mice, thus resulting in the inhibition of fibroblast proliferation and activation. Collectively, this study suggests that fibroblast VDR upregulation may be a self-protective response to limit fibroblast proliferation and activation during pulmonary fibrosis by suppressing the JAK1/STAT3/ER stress pathway.

## 1. Introduction

Idiopathic pulmonary fibrosis (IPF) is a chronic, fibrosing interstitial pneumonia of unknown etiology that is characterized by histologic and radiological features of usual interstitial pneumonia (UIP) [1]. The histopathological hallmark of IPF patients is the formation of fibrotic foci, consisting predominantly of activated myofibroblasts that synthesize and deposit excessive extracellular matrix (ECM) proteins at the site of epithelial cell loss [2]. Although pirfenidone and nintedanib are approved for the clinical treatment of IPF, both drugs have limited efficacy in preventing disease progression and improving quality of life and are also associated with tolerability issues [3]. Therefore, it is particularly urgent to further explore the pathogenesis of IPF and to find effective and safe treatments.

The Vitamin D receptor (VDR) is a nuclear receptor and performs its biological function by binding to its ligand, vitamin D [4]. VDR is known to be highly expressed in the lung [5]. Lung-specific overexpression of VDR has been shown to have anti-inflammatory effects in lung tissues [6,7]. Deletion of VDR leads to the destruction of tight and adherens junctions in the lung and results in premature emphysema due to increased matrix metalloproteinases and the formation of lymphoid aggregates. This indicates that VDR dysregulation is linked to the pathogenesis of both acute lung injury and chronic obstructive pulmonary disease [8,9,10,11]. Supplementation of vitamin D has been shown to attenuate pulmonary fibrosis in animal models [12,13,14]. However, whether dysregulation of VDR occurs and contributes to the development of pulmonary fibrosis remains largely unknown.

The endoplasmic reticulum (ER) is primarily required for protein folding and performing quality control for proteins [15]. An imbalance in the correct protein folding environment of the ER can lead to the accumulation of unfolded or misfolded proteins, resulting in ER stress, which leads to the activation of the unfolded protein response (UPR) [15]. ER stress/UPR has been linked to the pathogenesis of IPF through the regulation of alveolar epithelial cell apoptosis, epithelial–mesenchymal transition, myofibroblast differentiation, and M2 macrophage polarization [15,16]. 

In this study, we first performed bioinformatics analysis using both bulk RNA-seq/microarrays and single-cell RNA sequencing data from UIP/IPF patients and bleomycin-induced murine pulmonary fibrosis models. We showed, for the first time, that VDR was specifically upregulated in lung fibroblasts during pulmonary fibrosis. Gene set enrichment analysis (GSEA) results indicated that UPR gene set activation was significantly enriched in lung fibroblasts obtained from UIP/IPF patients or bleomycin-treated mice. Therefore, the present study first investigated the effects of the stable VDR knockdown or VDR agonist on fibroblast proliferation and activation and then elucidated the effects of the vitamin D/VDR pathway on ER stress and the underlying mechanisms both in vitro and in vivo.

## 2. Materials and Methods

### 2.1. Animals

Eight-week-old male wild-type C57/BL/6 mice were purchased from Shanghai SLAC Laboratory Animal Co. (Shanghai, China) for this study. All animals were bred in an antigen- and virus-free room at a controlled temperature and had access to food and water ad libitum. All animal experiments were approved and conducted in compliance with the Animal Experimentation Ethics Committee of Shanghai University of Sport (Ethical approval number: 2015024; 2016014).

### 2.2. Administration of Bleomycin and Drug Treatment

After anesthesia, mice were treated with bleomycin (Selleck Chemicals, Houston, TX, USA) at a dose of 3 mg/kg in a total volume of 50 μL by a single intratracheal instillation [17]. Control mice received intratracheal instillations of 50 μL sterile PBS only. The VDR agonist paricalcitol (MCE, Princeton, NJ, USA) was dissolved in corn oil. To investigate the role of paricalcitol in bleomycin-induced lung fibrosis, mice were randomly divided into four groups: (1) Control group: mice were intratracheally instilled with PBS and injected intraperitoneally with vehicle. (2) Paricalcitol group: mice were intratracheally instilled with PBS and injected intraperitoneally with 750 ng/kg paricalcitol. (3) Bleomycin Group: mice were intratracheally instilled with bleomycin and injected intraperitoneally with vehicle. (4) Bleomycin and Paricalcitol: mice were intratracheally instilled with bleomycin and injected intraperitoneally with 750 ng/kg paricalcitol. Paricalcitol or vehicle was injected on day 2 after instillation of bleomycin, and the treatment was continued every other day until the end of the experiment [13,18]. This dose of paricalcitol is well tolerated and successfully activates VDR [19,20].

VDR-expressing lentiviral vector (Lv-VDR) and control lentiviral vector (Lv-NC) were purchased from Genechem Co., Ltd. (Shanghai, China). After anesthesia, C57/BL6 mice were intratracheally instilled with 4 × 10^7^ PFUs (in 50 µL) of Lv-VDR or Lv-NC [21]. Forty-eight hours later, mice were administered a single intratracheal dose of bleomycin at 3 mg/kg in a total volume of 50 μL. To investigate the role of intrapulmonary VDR overexpression in bleomycin-induced lung fibrosis, mice were randomly divided into 4 groups: (1) Lv-NC-group mice were intratracheally instilled with Lv-NC and PBS; (2) Lv-NC + Bleomycin-group mice were intratracheally instilled with Lv-NC and bleomycin; (3) Lv-VDR-group mice were intratracheally instilled with Lv-VDR and PBS; (4) Lv-VDR + Bleomycin-group mice were intratracheally instilled with Lv-VDR and bleomycin.

### 2.3. Pulmonary Function Test

Pulmonary function tests were performed using the DSI Buxco-PFT system (DSI, St. Paul, MN, USA). The mice were anesthetized to ensure that they were not breathing spontaneously or independently during the experiment. They were placed in a supine position, and a midline incision was made to insert a tracheal cannula, which was secured with a sterile suture. The mice were then transferred to a chamber for testing. We obtained the dynamic compliance and the forced vital capacity results from the Resistance & Compliance Test and the Pressure Volume Test, respectively [22].

### 2.4. Lung Masson’s Trichrome Staining

Masson’s trichrome staining was performed to analyze collagen deposition. The left lower lobes of the lungs were perfused and fixed with 4% paraformaldehyde and then processed for 4 µm paraffin sections. Masson’s trichrome staining (Servicebio, Wuhan, China) was performed according to the manufacturer’s instructions as previously described [17,23]. 

### 2.5. Analysis of Bulk RNA Profiling Data and Single Cell RNA Sequencing Data

The bulk RNA profiling data and single-cell RNA sequencing (scRNAseq) data were obtained from the NCBI Gene Expression Omnibus (GEO, 25 November 2021, https://www.ncbi.nlm.nih.gov/geo/, accessed on 20 November 2021). For the analysis of VDR expression in fibrotic lung tissues, we analyzed eight GEO datasets (GSE21369 [24], GSE53845 [25], GSE38958 [26,27], GSE110147 [28], GSE2052 [29,30,31], GSE101286 [32], GSE72073 [33], and GSE48149 [34,35]) of UIP/IPF patients and twelve GEO datasets (GSE103511 [36], GSE94522, GSE77326, GSE43695 [37], GSE25640 [38], GSE16846 [39], GSE37635 [40], GSE40151 [41], GSE42301 [42], GSE97825 [43], GSE112827 [43], and GSE180750) of murine models of bleomycin-induced pulmonary fibrosis. For the analysis of VDR expression in lung fibroblasts, we analyzed three GEO datasets (GSE135456, GSE135099, GSE40839) of primary isolated lung fibroblasts from UIP/IPF patients, two GEO datasets (GSE83657 and GSE161322) of primary isolated lung fibroblasts from bleomycin-treated mice, and three GEO datasets (GSE1724, GSE135099, and GSE183345) of TGFβ-treated lung fibroblasts. Additionally, a published scRNAseq dataset (GSE132771) was analyzed to compare the expression of VDR in all cell types or lung fibroblasts isolated from IPF and control samples. A foldChange (FC) ≥ 2 or FC ≤ 0.5 (|log2fold change| ≥ 1) and *p* value < 0.05 were set as the threshold for significant differential expression.

### 2.6. Lung Fibroblast Isolation

Primary mouse lung fibroblasts were isolated from male C57BL/6 mice, as described in the previous study [44]. Briefly, after anesthesia, the right ventricle of each mouse was injected with PBS to clear blood from the lungs. Under aseptic conditions, the lung lobe was placed in a Petri dish containing DMEM (Gibco, San Diego, CA, USA) and chopped, and then it was digested with 0.5 mg/mL trypsin. The cells were precipitated by centrifugation and cultured in a complete medium containing 10% FBS (Gibco, USA) and 1% penicillin/streptomycin (Gibco, USA) for 6 h. The non-adherent cells were removed, and the adherent cells were trypsinized and plated in supplemented DMEM. Lung fibroblasts at passage 4 were used, and these cells were characterized by FSP-1 staining. It was found that approximately 90% of the cells showed positive staining.

### 2.7. Cell Culture and Stable Transfection

The mouse lung epithelial (MLE-12) cells from Fuheng Biotechnology Co. (Shanghai, China) were cultured in DMEM/F12 (Gibico) supplemented with 10% FBS (Gibco, USA), 1% penicillin/streptomycin (Gibco, USA), insulin-transferrin-sodium selenite (Sigma, USA), hydrocortisone 10 nM (Gibco, USA), β-estradiol 10 nM (Gibco, USA), 10 mM HEPES (Gibco, USA), and 2 mM L-glutamine (Gibco, USA). 

Human embryonic lung fibroblasts (MRC-5), obtained from Fuheng Biotechnology Co. (Shanghai, China), were cultured in minimum essential medium (Gibco, USA) containing 10% FBS (Gibco, USA), 1% penicillin/streptomycin (Gibco, USA), 1% non-essential amino acids (Gibco, USA), and 1% sodium pyruvate solution (Gibco, USA) at 37 °C in a 5% CO_2_ atmosphere [44].

The mouse lung fibroblast Mlg cells were purchased from Jennio Biotech Co. (Guangzhou, China) and cultured in DMEM/F12 (Gibco, USA) medium containing 10% FBS (Gibco, USA) and 1% penicillin/streptomycin (Gibco, USA). To achieve a stable knockdown of VDR expression, Mlg cells were transfected with shRNA lentivirus targeting VDR (Lv-ShRNA-VDR) or scrambled shRNA lentivirus (Lv-ShRNA-NC), which were designed and synthesized by Shanghai GeneChem Co. (Shanghai, China). Briefly, Mlg cells were plated in 6-well plates at a density of 1 × 10^5^ cells/well one day prior to lentivirus infections. Then, following the reference [45] and the manufacturer’s instructions, Mlg cells were transfected with lentivirus vectors at a multiplicity of infection (MOI) of 50 in a serum-free medium. After 16 h of incubation, the serum-free medium was removed and replaced with a fresh medium containing 10% FBS. After 72 h of transfection, Mlg were treated with 2 μg/mL puromycin (MCE, USA) to generate a stable VDR-knockdown cell line according to the manufacturer’s instructions. The knockdown efficiency of Lv-ShRNA-VDR was confirmed by quantitative polymerase chain reaction and Western blot analysis. 

### 2.8. Cell Proliferation Assay 

Cell viability was determined using a MTT Cell Proliferation and Cytotoxicity Assay Kit (Beyotime, China) following the manufacturer’s instructions. Briefly, after treatment, cells were incubated in 10 µL MTT solution (5 mg/mL) at 37 °C for 4 h. The supernatants were then removed, and the resulting MTT formazan was dissolved in 100 µL of formazan dissolving solution for 4 h at 37 °C. Absorbance at 570 nm was measured using a microtiter plate reader (BioTek, Winooski, VT, USA).

The proliferation of cells was detected using EdU Cell Proliferation Kit with Alexa Fluor 555 (EpiZyme, Shanghai, China), which is based on incorporating the thymidine analog EdU (5-ethynyl-2′-deoxyuridine) into the DNA synthesis process. EdU was administered to the cell culture medium (10 μM, 2-h incubation) and tagged with Alexa Fluor 555 via a click reaction. Images were captured using a Nikon Fluorescent microscope and analyzed using Fiji software (https://imagej.net/software/fiji/downloads, accessed on 12 July 2023).

### 2.9. Quantitative Polymerase Chain Reaction (qPCR)

Total RNA was isolated with the QIAGEN RNA extraction kit, following the manufacturer’s instructions. cDNA was synthesized using PrimeScript™ RT reagent Kit with gDNA Eraser from Takara Biotechnology, China. qPCR was performed using ChamQ Universal SYBR qPCR Master Mix from Vazyme, China. The relative quantization of gene expression was determined using the comparative Ct (threshold cycle) method with arithmetic formulae (2^−ΔΔ^Ct). The primer sequences (5′→3′) are provided in Supplemental Table S1. A reference gene evaluation, including all qPCR samples and three potential reference targets (β-actin, GAPDH and α-tubulin), was conducted with GeNorm and Normfinder calculations [46,47]. The most stable reference gene expression was achieved for β-actin, which was used for normalization in this study.

### 2.10. Western Blot Analysis

Total proteins were extracted from lung tissues, primary fibroblasts, and Mlg cells using cold RIPA buffer containing protease and phosphatase inhibitor cocktail (Proteintech, Wuhan, China). The proteins were then separated with 10% SDS-PAGE and transferred to PVDF membranes (Millipore, Bedford, MA, USA). The membranes were blocked in 5% (*w*/*v*) non-fat skimmed milk/TBST for 1.5 h. Primary antibodies against α-SMA (1:500, Servicebio), Fibronectin (1:1000, Cell Signaling Technology, Danvers, MA, USA), Collagen-1 (1:1000, Cell Signaling Technology), Grp78 (1:1000, Cell Signaling Technology), CHOP (1:1000, Cell Signaling Technology), p-STAT3 (1:500, Affinity), p-JAK1 (1:1000, Cell Signaling Technology), STAT3 (1:1000, Proteintech), JAK1 (1:1000, Proteintech), VDR (1:1000, Proteintech), β-actin (1:3000, Sigma, St. Louis, MO, USA) and ATF6 (1:500, Affinity, Changzhou, China) were incubated with the membranes overnight at 4 °C. After washing three times with TBST buffer, the membranes were incubated in horseradish peroxidase-labeled secondary antibodies (1:1000, Proteintech) for 1 h at room temperature. The Western blots were subsequently detected using enhanced chemiluminescence (Biosharp, Hefei, China), and the chemiluminescent signals were quantified using Tanon Chemilumenescence Instrument (Tanon, Shanghai, China). The images were analyzed using Fiji software.

### 2.11. Immunofluorescence Staining

To assess α-SMA expression in Mlg, the cells were seeded on coverslips. After treatment, cells were washed with phosphate-buffered saline (PBS) and fixed in 4% paraformaldehyde for 20 min at room temperature. After blocking with goat serum for 1 hour, the cells were incubated with primary antibodies against α-SMA (1:500) at 4 °C overnight. The cells were then incubated with Alexa Fluor^®^ 488-conjugated secondary antibodies in the dark for 1 h. After counterstaining with 4′6-diamidino-2-phenylindole (DAPI), the cells were treated with Antifade Mounting Medium (Beyotime, China) to prevent fluorescence quenching. Images were captured using a Nikon Fluorescent microscope and analyzed using Fiji software.

For immunofluorescence staining of lung tissues, 4 μm paraffin-embedded sections were first deparaffinized and rehydrated. Antigen retrieval was performed by microwaving. After blocking with 10% normal goat serum, sections were incubated overnight at 4 °C with primary antibodies against Ki67 (Servicebio), FSP-1 (Servicebio), Grp78 (Servicebio), or α-SMA (Servicebio). Subsequently, lung sections were incubated with secondary antibodies conjugated with Alexa Fluor^®^ 647 or Alexa Fluor^®^ 488 for 1 h in the dark. Finally, nuclei were counterstained with DAPI. The fluorescent images were captured by Pannoramic MIDI (3D HISTECH, Budapest, Hungary). For quantification, five high-power fields were analyzed in lung tissue sections taken from each animal. The percentages of Ki67^+^/FSP-1^+^ cells in total FSP-1^+^ cells or α-SMA intensity were then determined.

### 2.12. Chromatin Immunoprecipitation Assay

ChIP assays were performed using the BeyoChIP™ ChIP Assay Kit (P2080S, Beyotime, Shanghai, China) according to the manufacturer’s instructions. Briefly, MRC-5 cells (1 × 10^6^) in a 10 cm culture dish were treated with 1% formaldehyde to cross-link chromatin-associated proteins to DNA. The cell lysates were subjected to ultrasound for 10 sets of 30 s pulses (Bioruptor Pico, Liege, Belgium) to shear the DNA into fragments of approximately 200 to 1000 bp. Equal cell lysates were incubated with 1 μg of primary antibody against STAT3 overnight (Proteintech, China), and anti-IgG antibody (Beyotime, China) was used as a negative control. All the chromatin supernatants were then incubated with 80 μL of Protein A/G Magnetic beads/Salmon Sperm DNA for 1 h at 4 °C with rotation. The protein-DNA complexes were reversed and purified to obtain pure DNA. The qPCR following ChIP was performed with the specific primers listed below: 5′-CACTGGCCGCTGAAATTTAA-3′ and 5′-CACATACATCACGAACTCC-3′ for the human ATF6 promoter region (−1020/−909); 5′-GGAACGCTGCTGCATTATGTA-3′ and 5′-ACGTATGCTTCTAGGACCA-3′ for the human ATF6 promoter region (−1175/−1050). 

### 2.13. Statistical Analysis

The data were presented as means ± SEM. Raw data were analyzed for normal distribution using the Kolmogorov–Smirnov test. If the data were normally distributed, they were compared using two-tailed unpaired *t*-tests or one-way analysis of variance (ANOVA), followed by a Student–Newman–Keuls post hoc test for two-group or multi-group comparisons, respectively. A *p* value of <0.05 was considered significant.

## 3. Results

### 3.1. VDR Was Specifically Upregulated in Lung Fibroblasts during Pulmonary Fibrosis

To investigate the role of VDR in the progression of pulmonary fibrosis, we first analyzed eight published GEO datasets for VDR expression in lung tissues from UIP/IPF patients. As shown in Supplemental Table S2, VDR expression levels were not significantly changed in lung tissue samples from UIP/IPF patients compared with control samples. We then analyzed 12 published GEO datasets for VDR expression in lung tissues from murine models of bleomycin-induced pulmonary fibrosis. As shown in Supplemental Table S3, VDR expression levels were not significantly changed in lung tissue samples from mice exposed to bleomycin for 4 days to 4 months compared with those from control mice.

**Figure 1:**
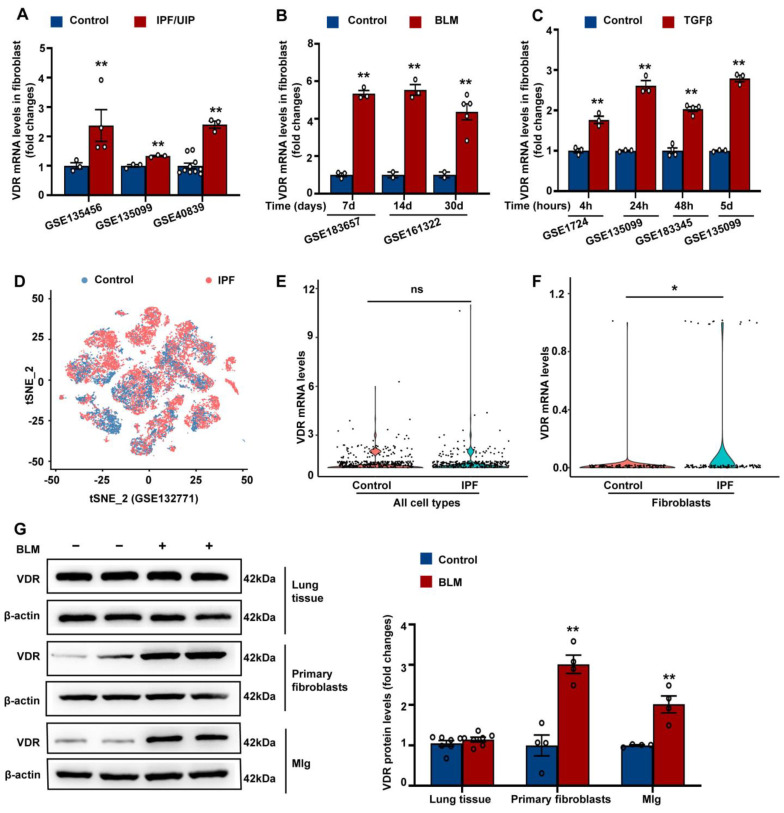
VDR was specifically upregulated in lung fibroblasts during pulmonary fibrosis. (**A**,**B**) Published GEO datasets were analyzed for VDR expression in lung fibroblasts isolated from UIP/IPF patients (**A**) or bleomycin-treated mice (**B**). (**C**) Published GEO datasets were analyzed for VDR expression in primary cultured lung fibroblasts treated with TGF-β in vitro for the indicated exposure times. (**D**–**F**) A published scRNA-seq dataset (GSE132771) was analyzed in this study. (**D**) shows UMAP plot of all cells acquired by scRNA-seq of human samples. Cells were obtained from normal lungs (blue) and IPF lungs (red). When all cell types were analyzed as a pool, no significant difference was found in VDR levels between control and IPF groups (**E**). However, VDR expression was significantly elevated in fibroblasts from the lungs of patients with IPF (**F**). (**G**) Mice were intratracheally instilled with bleomycin (3 mg/kg) or saline. Lung tissues or fibroblasts were obtained at day 14 after bleomycin instillation. The mouse lung fibroblast cell line Mlg was treated with bleomycin (5 μg/mL) for 48 h. VDR protein expression in lung tissues, lung fibroblasts, and Mlg cells was assessed by Western blot. Representative protein bands are presented on the left side of corresponding histograms. Lung tissue samples were collected from 7 control mice and 7 bleomycin-treated mice. All cell samples were collected from four independent experiments. Data are expressed as means ± SEM. * *p* < 0.05, ** *p* < 0.01 vs. Control, ns represents non-significant. BLM represents bleomycin.

Lung fibroblasts are known to express functional vitamin D receptors [48]. We analyzed published GEO datasets to determine the expression of VDR in lung fibroblasts isolated from UIP/IPF patients or bleomycin-treated mice. As shown in Figure 1A,B, VDR expression was significantly higher in lung fibroblasts isolated from UIP/IPF patients or bleomycin-treated mice compared to those isolated from control patients or mice, respectively. Additionally, we analyzed published GEO datasets for VDR expression in primary cultured lung fibroblasts treated with TGF-β in vitro. It was found that TGF-β treatment for 4 h to 5 days significantly increased VDR expression in both human and murine lung fibroblasts (Figure 1C). 

We then analyzed a published scRNA-seq data (GSE132771) to compare the expression of VDR in IPF and control samples (Figure 1D). This dataset collected CD235a (erythrocyte marker) cells as all lung cells. By analyzing representative markers, we identified T cells, B cells, dendritic cells, epithelial cells, endothelial cells, macrophages, monocytes, neutrophils, NK cells, and fibroblasts (Supplementary Figure S1). As shown in Figure 1E, when all cell types were analyzed as a pool, no significant difference was found in VDR levels between control and IPF groups. However, VDR expression was significantly elevated in fibroblasts from the lungs of patients with IPF (Figure 1F).

To validate the results of bioinformatic analysis, we then examined VDR protein expression in lung tissues and lung fibroblasts obtained from mice intratracheally exposed to bleomycin or saline for 14 days. As shown in Figure 1G, VDR expression in lung fibroblasts, but not lung tissues, was significantly increased in bleomycin-exposed mice compared to control mice. Additionally, we also found that bleomycin treatment significantly increased VDR protein expression in mouse lung fibroblast cell line Mlg cells. Taken together, the bioinformatic analysis and validation results indicated that VDR is specifically upregulated in lung fibroblasts during pulmonary fibrosis.

### 3.2. Stable Knockdown of VDR Promotes Lung Fibroblast Proliferation and Activation

**Figure 2:**
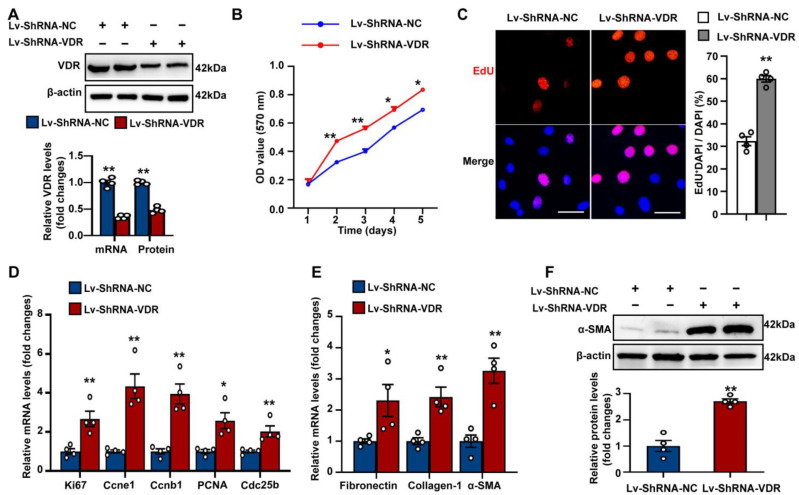
Stable knockdown of VDR promotes lung fibroblast proliferation and activation. Mlg cells were transfected with shRNA lentivirus targeting VDR (Lv-ShRNA-VDR) to establish a fibroblast cell line with stable VDR knockdown. Control cells were transfected with scrambled shRNA lentivirus (Lv-ShRNA-NC). (**A**) The knockdown efficiency of stable transfection of Lv-ShRNA-VDR or Lv-ShRNA-NC was confirmed by measuring mRNA and protein levels of VDR by qPCR and Western blot analysis, respectively. (**B**) Cell proliferation was assessed using the MTT assay at the indicated time point. (**C**), Cell proliferation was also assessed using the EdU incorporation assay. The percentage of proliferative cells is shown on the right of the representative images as a ratio of EdU^+^ cells to the total cell number. Scale bars correspond to 50 μm. (**D**,**E**) qPCR was used to determine mRNA levels of the pro-proliferative genes (**D**) and fibrosis-specific genes (**E**). (**F**) Western blot analysis was used to determine the protein expression of α-SMA. Representative protein bands are shown on the top of corresponding histograms. Data are expressed as means ± SEM (n = 4). * *p* < 0.05, ** *p* < 0.01 vs. Lv-ShRNA-NC.

Fibroblast activation plays a central role in the initiation and maintenance of fibrotic lesions in pulmonary fibrosis. To investigate the role of VDR in controlling fibroblast proliferation and activation, we established stable VDR knockdown in Mlg cells by using lentiviral vectors expressing short hairpin RNA (shRNA). The effectiveness of VDR knockdown in Mlg cells was confirmed through Western blot analysis and qPCR (Figure 2A). Both the MTT cell viability assay and the EdU incorporation assay demonstrated that stable knockdown of VDR significantly increased the proliferation of lung fibroblasts (Figure 2B,C). Additionally, mRNA expression of the pro-proliferative genes Ki67, Ccne1, Ccnb1, proliferating cell nuclear antigen (PCNA), and Cdc25b was significantly increased upon VDR knockdown, as shown in Figure 2D. 

In addition, we observed that the stable knockdown of VDR profoundly increased the mRNA expression of fibrosis-specific genes fibronectin, collagen-1, and α-SMA in Mlg fibroblasts (Figure 2E). Western blot analysis further confirmed the upregulation of α-SMA in VDR-knockdown fibroblasts compared with control cells, suggesting fibroblast-to-myofibroblast transdifferentiation (Figure 2F). Collectively, these findings suggest that VDR knockdown alone can promote cell proliferation and activation in lung fibroblasts.

### 3.3. VDR Agonist Suppresses Lung Fibroblast Proliferation and Activation In Vitro

**Figure 3:**
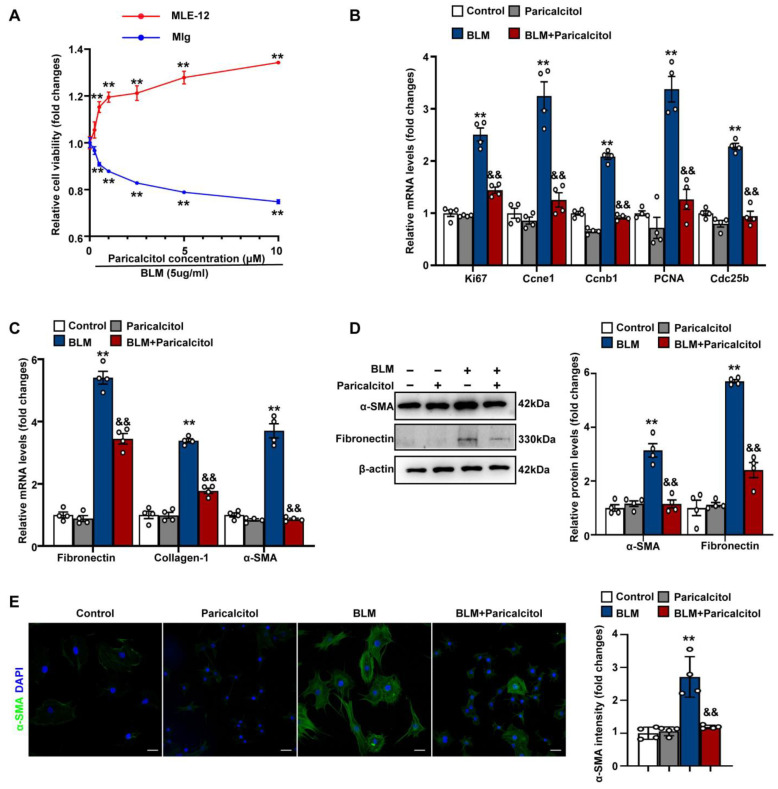
VDR agonist suppresses lung fibroblast proliferation and activation in vitro. (**A**) Mlg and MLE-12 cells were treated with bleomycin (5 μg/mL) in the presence of increasing concentrations of paricalcitol (0~10 μM) for 48 h. Cell proliferation was detected by MTT Assay. (**B**–**E**) Mlg cells were treated with saline or bleomycin (5 μg/mL) in the presence of vehicle or paricalcitol (2.5 μM) for 48 h. (**B**,**C**) qPCR was used to determine mRNA levels of the pro-proliferative genes (**B**) and fibrosis-specific genes (**C**). (**D**) Protein levels of α-SMA and fibronectin were determined by Western blot analysis. Representative protein bands are presented on the left of corresponding histograms. (**E**) Mlg cells were stained with primary antibody against the myofibroblast marker α-SMA (green). Nuclei were stained with blue using 4′,6-diamidino-2-phenylindole (DAPI). Quantification of the α-SMA mean intensity in fibroblasts are presented on the right of the representative images. Scale bars correspond to 50 μm. Data are expressed as means ± SEM (n = 4). ** *p* < 0.01 vs. Control, && *p* < 0.01 vs. BLM. BLM represents bleomycin.

We next observed the effect of VDR agonist paricalcitol on lung fibroblast proliferation and activation. Mlg cells were treated with increasing concentrations of paricalcitol (0.1~10 μM) in the presence of bleomycin (5 μg/mL) for 48 h. It was found that paricalcitol significantly inhibited Mlg cell proliferation in a dose-dependent manner (Figure 3A). In contrast, paricalcitol at the doses of 0.1~10 μM dose-dependently increased cell proliferation in lung epithelial cell line MLE-12 cells, which was consistent with previous studies [49]. Additionally, treatment with paricalcitol at a dose of 2.5 μM for 48 h significantly decreased EdU positive proliferative Mlg cells while increasing the number of EdU-positive proliferative MLE-12 cells in the presence of bleomycin (Supplementary Figure S2A). These data indicated that VDR agonist selectively inhibits cell proliferation of lung fibroblasts, but not epithelial cells.

As shown in Figure 3B,C, paricalcitol at a dose of 2.5 μM significantly inhibited bleomycin-induced increases in mRNA expression of the pro-proliferative genes Ki67, PCNA and Ccne1, Ccnb1, Cdc25b, as well as fibrosis-specific genes fibronectin, collagen-1 and α-SMA in Mlg fibroblasts. Western blot analysis further confirmed the inhibitory effects of paricalcitol on bleomycin-induced protein expression of fibronectin and α-SMA in lung fibroblasts (Figure 3D). Immunofluorescence staining also showed a significant reduction in α-SMA expression in Mlg cells treated with paricalcitol plus bleomycin compared to cells treated with bleomycin alone, suggesting the inhibitory effect of paricalcitol on bleomycin-induced fibroblast-to-myofibroblast transdifferentiation (Figure 3E). Collectively, these findings suggest that VDR agonist suppresses bleomycin-induced lung fibroblast proliferation and activation.

### 3.4. VDR Agonist Suppresses, Whereas Stable Knockdown of VDR Stimulates Endoplasmic Reticulum Stress through Regulation of JAK/STAT3 Signaling Pathway in Lung Fibroblasts

**Figure 4:**
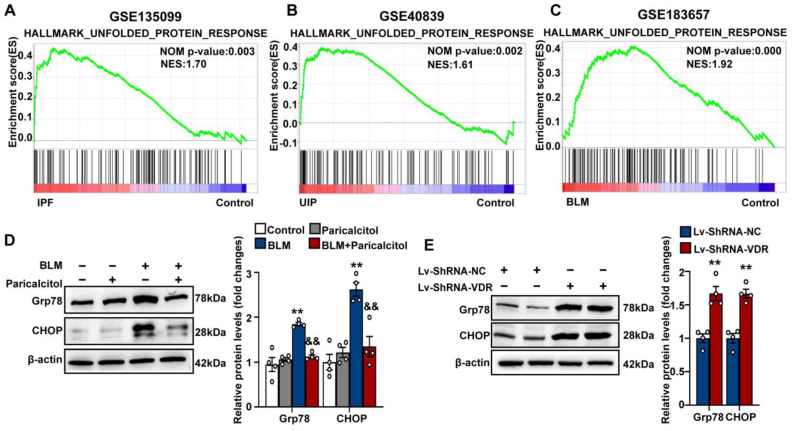
VDR agonist suppresses while stable knockdown of VDR stimulates endoplasmic reticulum stress in lung fibroblasts. (**A**–**C**) Following Hallmark analysis of GSEA, the gene set of unfolded protein response (UPR) was found to be enriched in lung fibroblasts obtained from IPF patients (**A**), UIP patients (**B**), and bleomycin-treated mice (**C**). (**D**) Mlg cells were treated with saline or bleomycin (5 μg/mL) in the presence of vehicle or paricalcitol (2.5 μM) for 48 h. Protein levels of Grp78 and CHOP were determined by Western blot analysis. Representative protein bands are presented on the left of corresponding histograms. Data are expressed as means ± SEM (n = 4). ** *p* < 0.01 vs. Control. && *p* < 0.01 vs. BLM. BLM represents bleomycin. (**E**) Mlg cells were stably transfected with Lv-ShRNA-NC or Lv-ShRNA-VDR. Protein levels of Grp78 and CHOP were determined by Western blot analysis. Representative protein bands are presented on the left of corresponding histograms. Data are expressed as means ± SEM (n = 4). ** *p* < 0.01 vs. Lv-ShRNA-NC.

To gain insights into the mechanisms responsible for the inhibitory effect of VDR on lung fibroblast proliferation and activation, gene set enrichment analysis (GSEA) was first performed to determine the enriched biological pathways in fibroblasts obtained from fibrotic lungs. Following a Hallmark analysis of GSEA, gene sets associated with well-known fibrosis-associated pathways including epithelial mesenchymal transition, inflammatory response, the TGF-β signaling, hypoxia, and apoptosis were significantly enriched in lung fibroblasts obtained from fibrotic lungs (Supplemental Figure S3). Notably, unfolded protein response (UPR), a process tightly related to endoplasmic reticulum (ER) stress, was also significantly enriched in lung fibroblasts obtained from UIP/IPF patients (GSE135099 and GSE40839, Figure 4A,B) or bleomycin-treated mice (GSE183657, Figure 4C). Although the UPR initially protects cells, it can also trigger apoptosis when ER stress is excessive and/or prolonged. Previous studies have shown that ER stress/UPR activation contributes to lung fibroblast proliferation and activation during pulmonary fibrosis [50,51]. However, whether VDR is involved in the regulation of ER stress in fibroblasts remains unknown. 

As shown in Figure 4D, we also detected increased protein expression of ER stress-related proteins Grp78 and the downstream transcript factor CHOP in bleomycin-treated Mlg fibroblasts compared with control cells, reflecting activation of ER stress. Treatment with VDR agonist paricalcitol profoundly blocked bleomycin-induced ER stress in lung fibroblasts, as evidenced by decreases in Grp78 and CHOP expression (Figure 4D). In contrast, stable knockdown of VDR resulted in significant increases in Grp78 and CHOP protein levels (Figure 4E). These findings indicate that VDR agonist suppresses in-lung fibroblasts, whereas stable knockdown of VDR stimulates ER stress.

**Figure 5:**
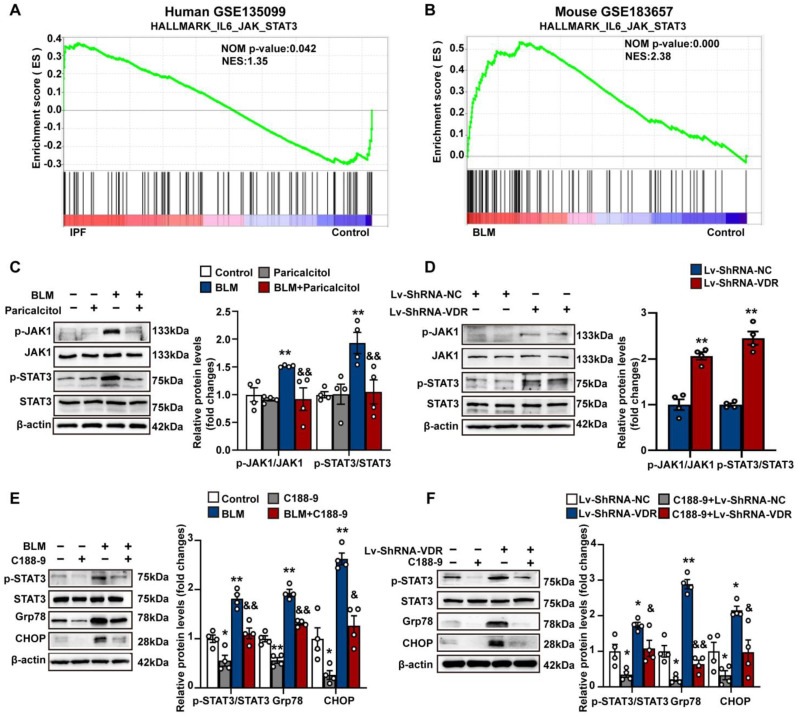
VDR agonist suppresses endoplasmic reticulum stress, while stable knockdown of VDR stimulates endoplasmic reticulum stress through regulation of JAK/STAT3 signaling pathway in lung fibroblasts. (**A**,**B**) Hallmark analysis of GSEA reveals that the IL6-JAK-STAT3 signaling pathway is enriched in lung fibroblasts obtained from IPF patients (**A**) and bleomycin-treated mice (**B**). (**C**) Mlg cells were treated with saline or bleomycin (5 μg/mL) in the presence of vehicle or paricalcitol (2.5 μM) for 48 h. Protein levels of phosphorylated JAK1, JAK1, phosphorylated STAT3, and STAT3 were determined by Western blot analysis. Representative protein bands were presented on the left of the histograms. Data are expressed as means ± SEM (n = 4). ** *p* < 0.01 vs. Control, && *p* < 0.01 vs. BLM. (**D**) Mlg cells were stably transfected with Lv-ShRNA-NC or Lv-ShRNA-VDR. Protein levels of phosphorylated JAK1, JAK1, phosphorylated STAT3, and STAT3 were determined by Western blot analysis. Representative protein bands are presented on the left of the histograms. Data are expressed as means ± SEM (n = 4). ** *p* < 0.01 vs. Lv-ShRNA-NC. (**E**) Mlg cells were treated with saline or bleomycin (5 μg/mL) in the presence of vehicle or STAT3 inhibitor C188-9 (4 μM) for 48 h. Protein levels of phosphorylated STAT3, STAT3, Grp78, and CHOP were determined by Western blot analysis. Representative protein bands were presented on the left of the histograms. Data are expressed as means ± SEM (n = 4). * *p* < 0.05, ** *p* < 0.01 vs. Control, & *p* < 0.05, && *p* < 0.01 vs. BLM. (**F**) Mlg cells stably transfected with Lv-ShRNA-NC or Lv-ShRNA-VDR were treated with vehicle or STAT3 inhibitor C188-9 (4 μM) for 48 h. Protein levels of phosphorylated STAT3, STAT3, Grp78, and CHOP were determined by Western blot analysis. Representative protein bands were presented on the left of the histograms. Data are expressed as means ± SEM (n = 4). * *p* < 0.05, ** *p* < 0.01 vs. Lv-ShRNA-NC. & *p* < 0.05, && *p* < 0.01 vs. Lv-ShRNA-VDR. BLM represents bleomycin.

To investigate the signaling pathways involved in the regulation of ER stress, we performed GSEA to determine the enriched signaling pathways in fibroblasts obtained from fibrotic lungs. Following Hallmark, Kyoto Encyclopedia of Genes and Genomes (KEGG) and Reactome pathway analysis of GSEA, we found that gene sets associated with ER stress/UPR-associated pathways, including the p53 pathway; PERK-regulated gene expression; and ATF4 activated genes in response to ER stress were significantly enriched in lung fibroblasts obtained from fibrotic lungs (Supplemental Figure S4). Intriguingly, we also observed that JAK/STAT signaling, a molecular pathway known to be activated in interstitial lung diseases, was also significantly enriched in lung fibroblasts obtained from IPF patients (GSE135099, Figure 5A) and bleomycin-treated mice (GSE183657, Figure 5B). However, it remains to be elucidated whether activation of the JAK/STAT pathway contributes to ER stress during the pathogenesis of pulmonary fibrosis remains.

As shown in Figure 5C,D, we also detected increased phosphorylation of JAK1 and STAT3 in both bleomycin-treated and stable VDR-knockdown Mlg fibroblasts compared with control cells, reflecting activation of the JAK/STAT3 signaling pathway. Treatment with the VDR agonist paricalcitol profoundly blocked bleomycin-induced phosphorylation of JAK1 and STAT3 in lung fibroblasts (Figure 5C). In addition, we found that C188-9, a novel small-molecule STAT3 inhibitor, not only reduced phosphorylated STAT3, but also significantly decreased Grp78 and CHOP protein levels in lung fibroblasts treated with bleomycin (Figure 5E). Stable VDR-knockdown-induced STAT3 phosphorylation and upregulation of Grp78 and CHOP were also blocked by C188-9 (Figure 5F). 

Taken together, these findings indicate that the VDR agonist suppresses, whereas stable knockdown of VDR stimulates ER stress through the regulation of the JAK/STAT3 signaling pathway in lung fibroblasts.

### 3.5. VDR Agonist Inhibits STAT3-Dependent ATF6 Transcription and Endoplasmic Reticulum Stress in Human Lung Fibroblasts

**Figure 6:**
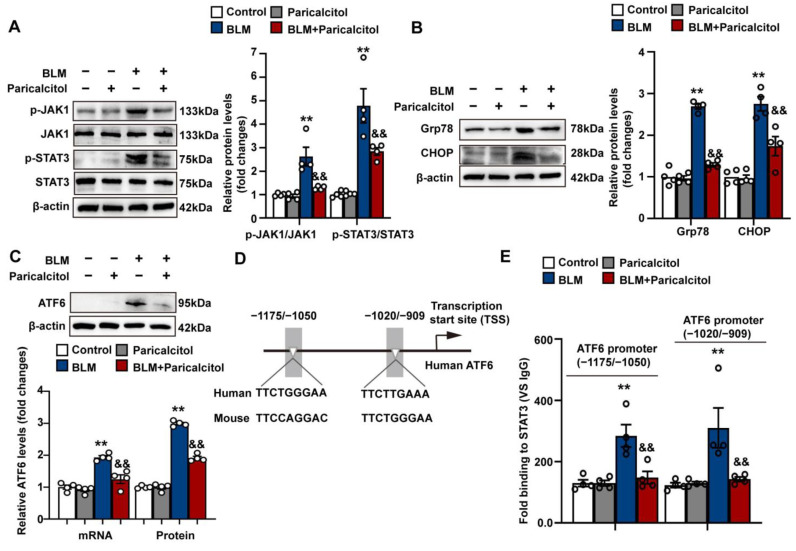
VDR agonist inhibits STAT3-dependent ATF6 transcription and endoplasmic reticulum stress in human lung fibroblasts. MRC-5 cells were treated with saline or bleomycin (5 μg/mL) in the presence of vehicle or paricalcitol (2.5 μM) for 48 h. (**A**) Protein levels of phosphorylated JAK1, JAK1, phosphorylated STAT3, and STAT3 were determined by Western blot analysis. Representative protein bands were presented on the left of the corresponding histograms. (**B**) Protein levels of Grp78 and CHOP were determined by Western blot analysis. Representative protein bands were presented on the left of corresponding histograms. (**C**) qPCR and Western blot analysis were used to determine mRNA and the protein levels of ATF6, respectively. Representative protein bands were presented on the top of corresponding histograms. (**D**) A schematic diagram shows two predicted conservative consensus STAT3-binding motifs in the proximal region of both human and mouse ATF6 promoters. (**E**) Chromatin immunoprecipitation (ChIP) assay showed enrichment of STAT3 on two consensus STAT3-binding motifs in human ATF6 promoter. Data are expressed as means ± SEM (n = 4). ** *p* < 0.01 vs. Control. && *p* < 0.01 vs. BLM. BLM represents bleomycin.

We further confirmed the effects of VDR activation on bleomycin-induced ER stress in human lung fibroblast cell line MRC-5 cells. As shown in Figure 6A,B, it was found that the VDR agonist paricalcitol also inhibited BLM-induced activation of JAK1/STAT3 and ER stress in MRC-5.

It has been well recognized that the UPR is controlled by three sensors, each activating distinct signaling cascades and transcription factors (TFs), including PKR-like ER kinase (PERK), inositol requiring 1α (IRE1α), and activating transcription factor 6 (ATF6) [15,16]. Notably, a recent study identified consensus STAT3 binding motifs on the promoter region of human ATF6 and confirmed that STAT3 functions to promote the transcription of the ATF6, which induces ER stress in human ovarian epithelial cancer cells [52]. We then investigated whether VDR activation might affect STAT3-dependent ATF6 transcription in fibroblasts. As shown in Figure 6C, the VDR agonist paricalcitol significantly inhibited bleomycin-induced mRNA and protein expression of ATF6 in MRC-5 cells, suggesting that VDR activation can regulate ATF6 expression at the transcriptional level. The potential biding sites of STAT3 on ATF6 promoter were then predicted using the JASPAR database (https://jaspar.genereg.net/, accessed on 12 July 2023). Two conservative consensus STAT3-binding motifs (TTMXXXDAA, D = A/G; M = A/C; X = any. −1175 to −1050 bp, and −1020 to −909 bp relative to the transcription start site of human ATF6) were found in the proximal region of both human and mouse ATF6 promoters (Figure 6D). Using conventional chromatin immunoprecipitation (ChIP)-qPCR assay, we found that bleomycin treatment significantly increased the binding of STAT3 to two consensus STAT3-binding motifs on the ATF6 promoter in MRC-5 cells, which was largely inhibited by the VDR agonist paricalcitol (Figure 6E). These findings suggest that VDR agonist inhibits STAT3-dependent ATF6 transcription in fibroblasts.

### 3.6. VDR Agonist Inactivates JAK/STAT3 Signaling Pathway and Suppresses Endoplasmic Reticulum Stress, Thus Resulting in Inhibition of Fibroblast Proliferation and Differentiation into Myofibroblast during Bleomycin-Induced Pulmonary Fibrosis

**Figure 7:**
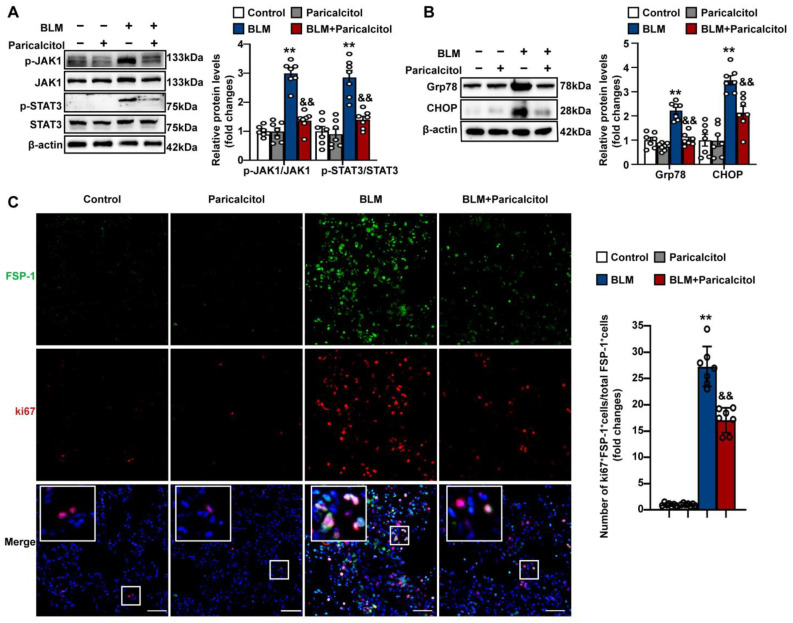
VDR agonist inactivates JAK/STAT3 signaling pathway and suppresses endoplasmic reticulum stress during bleomycin-induced pulmonary fibrosis. Mice were randomly divided into four groups: Control, Paricalcitol, BLM, and BLM + Paricalcitol. (**A**) Protein levels of phosphorylated JAK1, JAK1, phosphorylated STAT3, and STAT3 in lung tissues were determined in the Control, Paricalcitol, BLM, and BLM + Paricalcitol groups. Representative protein bands were presented on the left of the histograms. (**B**), Protein levels of Grp78 and CHOP in lung tissues were determined in the Control, Paricalcitol, BLM, and BLM + Paricalcitol groups. Representative protein bands were presented on the left of the histograms. (**C**) Lung sections were stained with fluorophore-labeled antibodies against the cell proliferation marker Ki67 (Alexa Fluor 647, red) and the fibroblast marker FSP-1 (Alexa Fluor 488, green). DAPI staining was used to detect nuclei (blue). The merge image represents double positive staining for FSP-1 and Ki67. Areas in white boxes were shown enlarged. Scale bars correspond to 50 μm. Quantification of the percentage of ki67^+^/FSP-1^+^ cells in total FSP-1^+^ cells in lung tissue sections is presented on the right of the representative images. Data are expressed as means ± SEM (n = 7). ** *p* < 0.01 vs. Control. && *p* < 0.01 vs. BLM. BLM represents bleomycin.

We then examined the effect of the VDR agonist paricalcitol on bleomycin-induced JAK/STAT3 activation and ER stress in vivo. It was found that paricalcitol treatment at a dose of 750 ng/kg for 2 weeks significantly reduced bleomycin-induced phosphorylation of JAK1 and STAT3 in the lung (Figure 7A). Additionally, paricalcitol treatment also reduced the upregulation of Grp78 and CHOP induced by bleomycin. These findings indicate that the VDR agonist can ameliorate the activation of the JAK1/STAT3 signaling pathway and suppress ER stress in lung tissue (Figure 7B).

**Figure 8:**
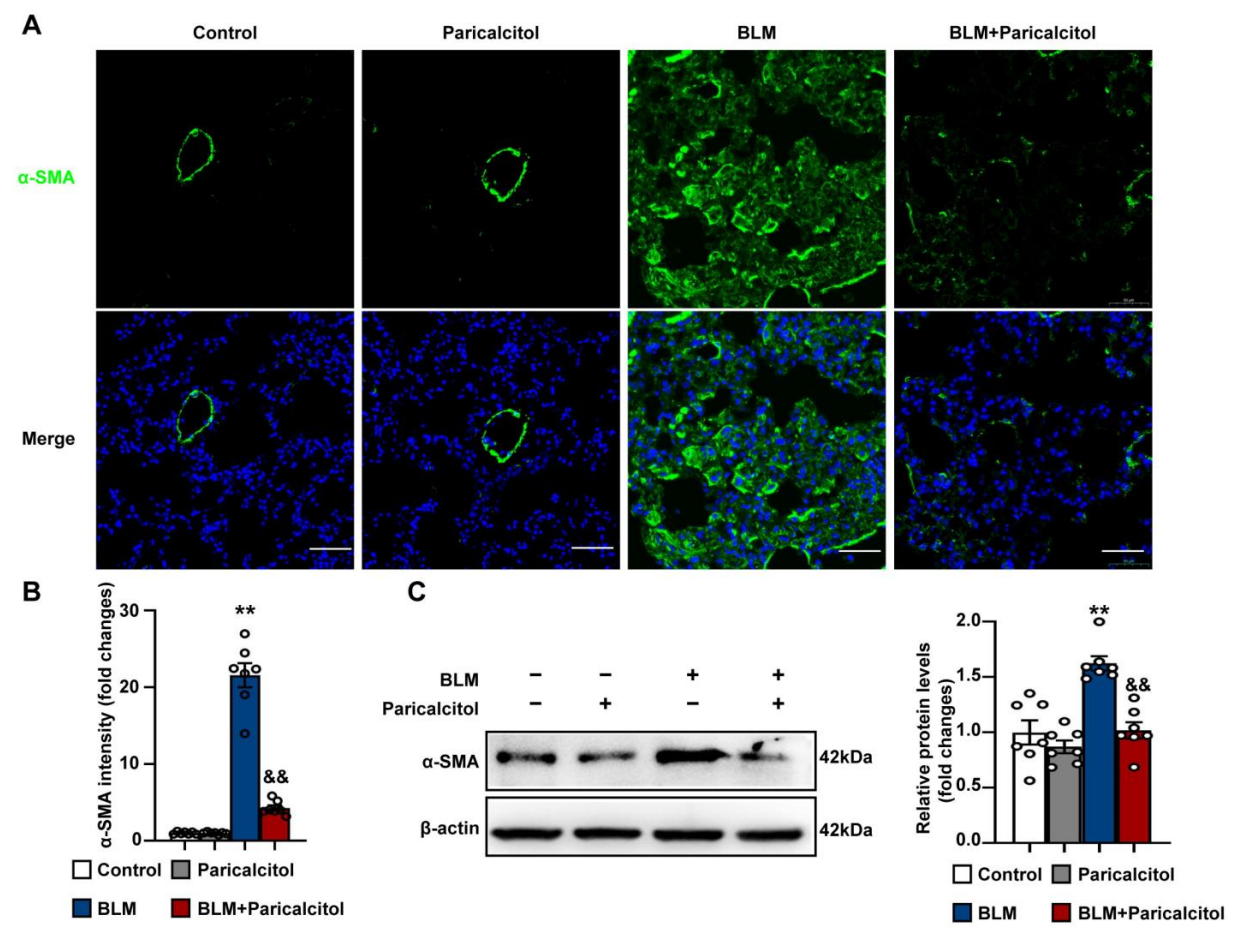
VDR agonist inhibits fibroblast differentiation into myofibroblast during bleomycin-induced pulmonary fibrosis. Mice were randomly divided into four groups: Control, Paricalcitol, BLM, and BLM + Paricalcitol. (**A**), Lung sections were stained with an anti-α-SMA antibody (green). DAPI staining was used to detect nuclei (blue). Scale bars correspond to 50 μm. (**B**) Quantification of the mean fluorescent intensity of α-SMA in lung tissue sections. (**C**) Protein levels of α-SMA in lung tissues were determined in the Control, Paricalcitol, BLM, and BLM + Paricalcitol groups. Representative protein bands were presented on the left of the histograms. Data are expressed as mean ± SEM (n = 7). ** *p* < 0.01 vs. Control. && *p* < 0.01 vs. BLM. BLM represents bleomycin.

We next determined whether paricalcitol inhibited lung fibroblast proliferation, as measured by double immunofluorescence staining against fibroblast marker FSP-1 and cell proliferation marker Ki67 in lung sections. As shown in Figure 7C, bleomycin-treated mice exhibited increased localization of FSP/Ki67 in lung tissues, which was significantly reduced by the administration of paricalcitol. Immunofluorescence staining against myofibroblast marker α-SMA was then performed on lung sections. It was found that bleomycin caused increased accumulation of α-SMA^+^ myofibroblasts, which was profoundly reduced by paricalcitol treatment (Figure 8A,B). The inhibitory effect of paricalcitol on bleomycin-induced α-SMA expression was also confirmed by Western blot analysis (Figure 8C). Collectively, these findings indicate that VDR activation results in the inhibition of fibroblast proliferation and differentiation into myofibroblasts during bleomycin-induced lung fibrosis.

### 3.7. VDR Agonist Attenuates Pulmonary Fibrosis and Improves Pulmonary Function in Mice Exposed to Bleomycin

**Figure 9:**
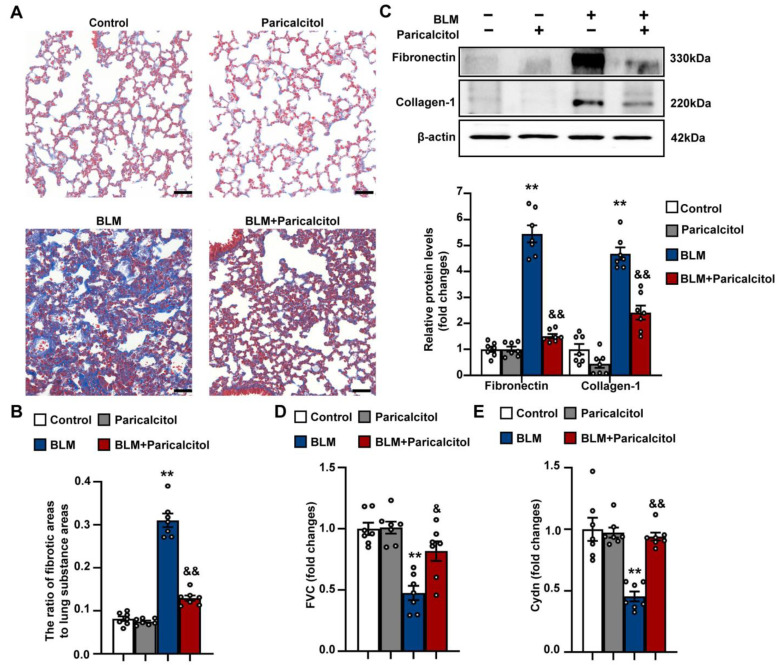
VDR agonist attenuates pulmonary fibrosis and improves pulmonary function in mice exposed to bleomycin. Mice were randomly divided into four groups: Control, Paricalcitol, BLM, and BLM + Paricalcitol. (**A**) Collagen deposition in lung tissues were examined by Masson’s trichrome staining in the Control, Paricalcitol, BLM, and BLM + Paricalcitol groups. Scale bars correspond to 50 μm. (**B**) Changes in the ratio of collagen-deposited areas to lung substance areas (a morphometric measure of pulmonary fibrosis). (**C**) Protein levels of fibronectin and collagen-1 in lung tissues were determined in the Control, Paricalcitol, BLM, and BLM + Paricalcitol groups. Representative protein bands were presented on the top of the histograms. (**D**,**E**) The forced vital capacity (FVC) and dynamic lung compliance (Cydn) were measured by Pressure Volume Test and Resistance & Compliance Test, respectively. Data are expressed as mean ± SEM (n = 7). ** *p* < 0.01 vs. Control. & *p* < 0.05, && *p* < 0.01 vs. BLM. BLM represents bleomycin.

We then examined the effect of VDR agonist on bleomycin-induced pulmonary fibrosis. As shown in Figure 9A,B, Masson’s trichrome staining revealed a significant increase in collagen deposition in the interstitium. The collagen deposition appeared diffusively in the lung parenchyma and was accompanied by epithelial thickening and cellular infiltrates. Treatment with paricalcitol at a dose of 750 ng/kg for 2 weeks markedly attenuated the collagen deposition and the destruction of normal pulmonary architecture in bleomycin-treated mice. Consistent with the histological evidence, paricalcitol treatment lso significantly reduced the bleomycin-induced upregulation of fibronectin and Collagen 1 (Figure 9C). 

Decreased forced vital capacity (FVC) and lung compliance are known to occur frequently in fibrotic lungs [22]. As shown in Figure 9D,E, bleomycin-treated mice exhibited significant reductions in both FVC and lung compliance compared to control mice, which was significantly reversed by the administration of paricalcitol for 2 weeks. These findings collectively indicate that the administration of a VDR agonist can attenuate pulmonary fibrosis and improve pulmonary function in mice exposed to bleomycin.

### 3.8. VDR Agonist Attenuates Pulmonary Fibrosis and Improves Pulmonary Function in Mice Exposed to Bleomycin

**Figure 10:**
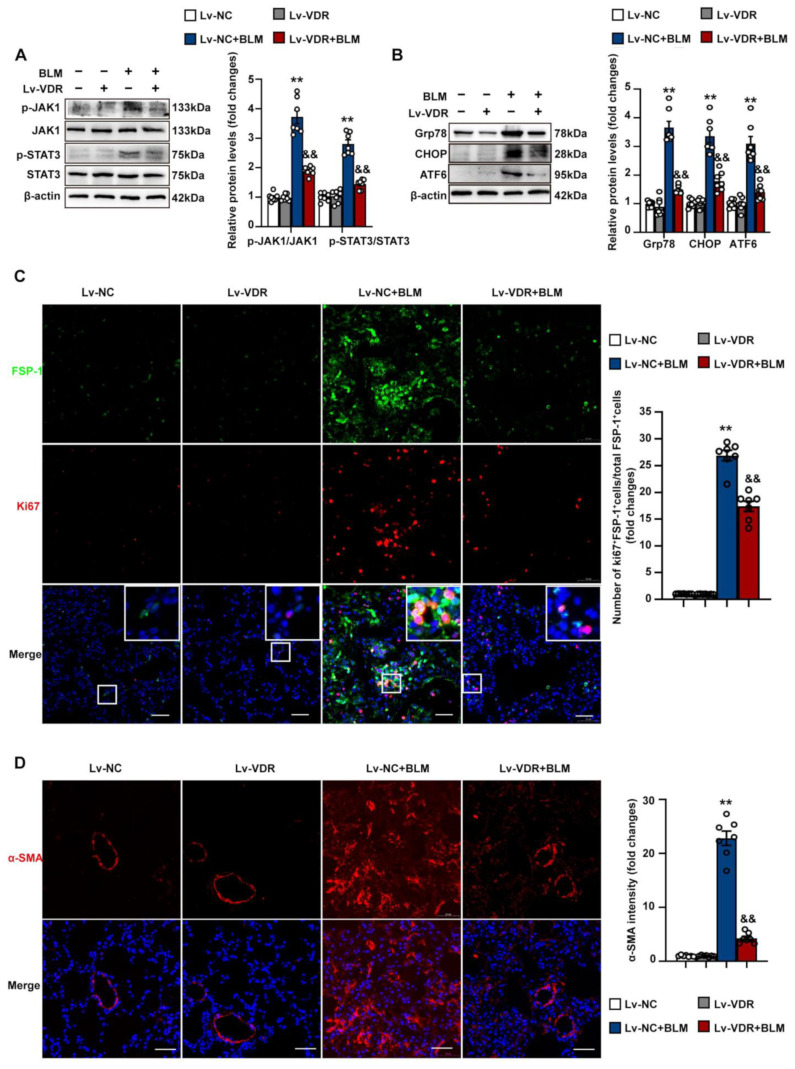
Intrapulmonary VDR overexpression inhibits JAK/STAT3 activation, endoplasmic reticulum stress, fibroblast proliferation and differentiation into myofibroblasts. Mice were randomly divided into four groups: Lv-NC, Lv-VDR, Lv-NC + BLM, and Lv-VDR + BLM. (**A**) The protein levels of phosphorylated JAK1, JAK1, phosphorylated STAT3, and STAT3 in lung tissues were determined by Western blot analysis. Representative protein bands were presented on the left of the histograms. (**B**) Protein levels of Grp78, CHOP and ATF6 in lung tissues were determined by Western blot analysis. Representative protein bands were presented on the left of the histograms. (**C**) Lung sections were stained with fluorophore-labeled antibodies against cell proliferation marker Ki67 (Alexa Fluor 647, red) and fibroblast marker FSP-1 (Alexa Fluor 488, green). DAPI staining was used to detect nuclei (blue). The merge image represents double positive staining for FSP-1 and Ki67. Areas in white boxes are shown enlarged. Scale bars correspond to 50 μm. Quantification of the percentage of Ki67^+^/FSP-1^+^ cells in total FSP-1^+^ cells in lung tissue sections are presented on the right of the representative images. (**D**) Lung sections were stained with anti-α-SMA antibody (red). DAPI staining was used to detect nuclei (blue). The mean fluorescent intensity of the α-SMA in lung tissue sections was quantified and presented on the right of the representative images. Scale bars correspond to 50 μm. Data are expressed as means ± SEM (n = 7). ** *p* < 0.01 vs. Control. && *p* < 0.01 vs. BLM. BLM represents bleomycin.

To further confirm the role of VDR activation on pulmonary fibrosis, we constructed a lentiviral vector (Lv-VDR) that expresses VDR and examined the effect of ectopic VDR overexpression on bleomycin-induced activation of JAK/STAT3 signaling pathway, ER stress, fibroblast proliferation, and differentiation into myofibroblasts in vivo. As shown in Supplementary Figure S5A, the intratracheal instillation of Lv-VDR led to an approximately 2.1-fold increase in pulmonary VDR expression. It was found that intrapulmonary VDR overexpression significantly reduced bleomycin-induced phosphorylation of JAK1 and STAT3 in the lung (Figure 10A). Additionally, the upregulation of Grp78, CHOP, and ATF6 induced by bleomycin was also reduced by Lv-VDR treatment (Figure 10B). To further elucidate the effect of VDR overexpression on bleomycin-induced fibroblast ER stress, double immunofluorescence staining against the fibroblast marker FSP-1 and Grp78 was performed in lung sections. As shown in Supplementary Figure S5B, bleomycin-treated mice exhibited an increased localization of FSP-1/Grp78 in lung tissues, which was significantly reduced by the intrapulmonary instillation of Lv-VDR. These findings indicate that intrapulmonary VDR overexpression can ameliorate bleomycin-induced activation of the JAK1/STAT3 signaling pathway and suppress ER stress in lung tissue.

We next determined whether intrapulmonary VDR overexpression affected lung fibroblast proliferation. As shown in Figure 10C, the intrapulmonary instillation of Lv-VDR significantly reduced the co-localization of FSP-1/Ki67 in lung tissues of bleomycin-treated mice. Immunofluorescence staining against the myofibroblast marker α-SMA showed that the bleomycin-induced accumulation of α-SMA^+^ myofibroblast was profoundly reduced by Lv-VDR treatment (Figure 10D). Collectively, these findings indicate that VDR overexpression results in the inhibition of fibroblast proliferation and differentiation into myofibroblasts during bleomycin-induced lung fibrosis. 

**Figure 11:**
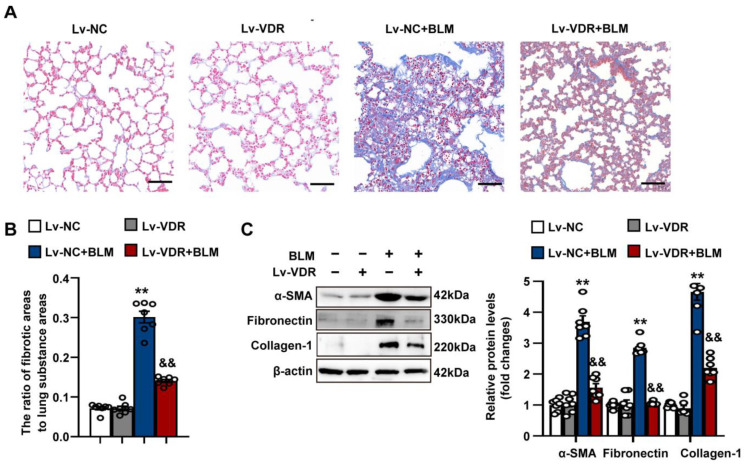
VDR overexpression attenuates pulmonary fibrosis in mice exposed to bleomycin. Mice were randomly divided into four groups: Lv-NC, Lv-VDR, Lv-NC + BLM, and Lv-VDR + BLM. (**A**) Collagen deposition in lung tissues was examined by Masson’s trichrome staining. Scale bars correspond to 50 μm. (**B**) Changes in the ratio of collagen-deposited areas to lung substance areas (a morphometric measure of pulmonary fibrosis). (**C**) Protein levels of α-SMA, fibronectin and collagen-1 in lung tissues were determined by Western blot analysis. Representative protein bands are presented on the left of the histograms. Data are expressed as mean ± SEM (n = 7). ** *p* < 0.01 vs. Control. && *p* < 0.01 vs. BLM. BLM represents bleomycin.

Furthermore, we found that intrapulmonary VDR overexpression significantly attenuated the collagen deposition and the destruction of pulmonary architecture in bleomycin-treated mice (Figure 11A,B). The bleomycin-induced upregulation of fibronectin, collagen-1 and α-SMA was significantly decreased by intrapulmonary VDR overexpression (Figure 11C). These results indicate that VDR ectopic expression attenuates pulmonary fibrosis in mice exposed to bleomycin. 

## 4. Discussion

Vitamin D is known to exert its principle biological functions through binding to VDR, a ligand-dependent transcriptional factor [14]. Deficiency of vitamin D has been implicated in the development of interstitial lung diseases. Low serum vitamin D values are observed in IPF patients [53,54]. In addition, serum vitamin D levels correlate with clinical and functional indicators of disease severity and are highly predictive of all-cause mortality in patients with IPF [53]. In lung fibrosis animal models, accumulating studies demonstrate that vitamin D deficiency aggravates, whereas supplementation of vitamin D improves pulmonary fibrosis of various etiology [12,13]. VDR is known to be highly expressed in the lung and plays critical roles in the regulation of inflammatory responses and pulmonary epithelial barrier integrity [8,9,10,11]. However, the role of VDR in the pathogenesis of pulmonary fibrosis remains largely unknown. In the present study, bioinformatics analysis was performed using both bulk RNA-seq/microarrays and single-cell RNA sequencing data from UIP/IPF patients. We showed for the first time that VDR expression was not changed in lung tissues of UIP/IPF patients. However, lung fibroblasts isolated from UIP/IPF patients exhibited higher VDR expression than those isolated from control samples. Bioinformatics analysis of bulk RNA-seq/microarrays and single-cell RNA sequencing data from bleomycin-induced murine pulmonary fibrosis models also mirrored the results observed in UIP/IPF patients. 

To the best of our knowledge, only one previously published study reported that VDR mRNA expression was downregulated in the lung tissues of bleomycin-treated mice and TGF-β-treated mouse lung fibroblasts [53]. However, our integrated bioinformatics analysis, using twelve GEO datasets of murine models of bleomycin-induced pulmonary fibrosis, two GEO datasets of lung fibroblasts isolated from bleomycin-treated mice, and one GEO dataset of single-cell RNA-seq of bleomycin-treated mice, suggests that VDR is specifically upregulated in lung fibroblasts during bleomycin-induced pulmonary fibrosis. This finding is further validated by our Western blot results. Additionally, three GEO datasets of TGF-β-treated lung fibroblasts also suggested the TGF-β-induced upregulation of VDR. Despite the contradictory results regarding the expression changes of VDR during bleomycin-induced lung fibrosis, previous studies have shown that vitamin D treatment attenuates fibrotic changes in lung fibrosis models induced by obesity, bleomycin and paraquat [13,14,55], which is consistent with our study. Notably, vitamin D has been shown to inhibit the TGF-β-induced pro-fibrotic phenotype of lung fibroblasts [48,56,57], while knockdown of VDR enhances the sensitivity of fibroblasts towards TGF-β [57]. In this study, we established stable VDR knockdown in Mlg cells by using shRNA-expressing lentiviral vectors and found that VDR knockdown alone promoted cell proliferation and fibroblast-to-myofibroblast transdifferentiation in lung fibroblasts. Taken together, these findings suggest that the upregulation of fibroblast VDR may be recognized as part of a novel self-protective response to regulate fibroblast proliferation and activation during pulmonary fibrosis.

ER stress has been linked to the development and progression of lung fibrosis through the regulation of epithelial cell apoptosis and epithelial-mesenchymal transition [15,16]. The ER stress regulation of PI3K/AKT signaling has been implicated in fibroblast proliferation and differentiation [51]. Inducing the fibroblast-specific deletion of thioredoxin domain containing 5, an ER resident chaperone, mitigates the progression of bleomycin-induced pulmonary fibrosis and lung function deterioration [50]. Recently, Ahmad et al. reported that Vitamin D ameliorates sepsis-induced acute lung injury via the downregulation of ER stress [58]. However, whether ER stress signaling contributes to the anti-fibrotic effects of vitamin D/VDR remains largely unknown. As expected, Hallmark GSEA analysis of three published GEO datasets showed that UPR, a process tightly related to ER stress, was significantly enriched in lung fibroblasts obtained from both UIP/IPF patients and bleomycin-treated mice. We found that the stable knockdown of VDR per se stimulated ER stress in lung fibroblasts, whereas the VDR agonist paricalcitol profoundly blocked ER stress. Additionally, both VDR agonist and intrapulmonary VDR overexpression suppressed ER stress in lung tissues exposed to bleomycin. Given the critical role of ER stress in fibroblast activation and fibrogenesis, our results suggest that the anti-fibrotic effects of vitamin D/VDR signaling may be at least partly due to the suppression of ER stress in lung fibroblasts.

Although ER stress has been shown to promote fibroblast proliferation and myofibroblast differentiation, limited studies have investigated the molecular mechanisms of ER stress induction in fibroblasts. Previous studies have demonstrated that the activation of PI3K/AKT promotes ER stress in lung fibroblasts [51,59]. PPP1R13B, a major member of the apoptosis-stimulating proteins of the p53 family, promotes the proliferation and migration of lung fibroblasts through the induction of ER stress [60]. 

The JAK/STAT molecular pathway is known to be activated under a variety of profibrotic/pro-inflammatory cytokines, thus contributing to the pathogenesis of pulmonary fibrosis. Enhanced expression of STAT3 in IPF fibroblasts is responsible for their fibrogenic phenotype by the regulation of collagen I secretion [61]. In addition, STAT3 also regulates IL-6–mediated and TGF-β1–mediated myofibroblast differentiation in both murine and human lung fibroblasts [62,63]. To date, there are no studies reporting the link between ER stress and JAK/STAT signaling pathway in the pathogenesis of pulmonary fibrosis; although, STAT3 activation is related to ER stress in hepatocytes and primary astrocytes during the acute-phase response and autoimmune encephalomyelitis, respectively [64,65]. In this study, our results demonstrated that the stable knockdown of VDR per se activated, whereas VDR agonist paricalcitol decreased the JAK1/STAT3 pathway in lung fibroblasts. Furthermore, JAK1/STAT3 phosphorylation in lung tissues exposed to bleomycin was blocked by either VDR agonist or intrapulmonary VDR overexpression. Moreover, we found that the STAT3 inhibitor significantly suppressed ER stress in lung fibroblasts induced by either bleomycin or the stable knockdown of VDR. Collectively, our study not only identifies JAK1/STAT3 as an upstream regulator of ER stress in fibroblasts, but also probes the mechanistic basis underlying the inhibitory effects of vitamin D/VDR on ER stress and fibroblast activation.

VDR activation has been shown to inactivate the JNK/STAT3 pathway in a variety of cell types. Notably, Català-Moll et al. recently demonstrated that VDR interacts with the phosphorylated form of STAT3 to form a complex with methylcytosine dioxygenase TET2 in dendritic cells with tolerogenic function (TolDC), thereby promoting TolDC-specific demethylation [66]. Additionally, a recent study identifies consensus STAT3 binding motifs on the promoter region of human ATF6 and confirms that STAT3 functions to promote the transcription of the ATF6, which induces endoplasmic reticulum stress in human ovarian epithelial cancer cells [52]. In the present study, we found two conservative consensus STAT3-binding motifs in the proximal region of both the human and mouse ATF6 promoter. The VDR agonist significantly inhibited the bleomycin-induced mRNA and protein expression of ATF6 in fibroblasts. Furthermore, ChIP-qPCR assay demonstrated that bleomycin treatment significantly increased the binding of STAT3 to two consensus STAT3-binding motifs on the ATF6 promoter in human lung fibroblast cell line MRC-5 cells, which was largely inhibited by VDR agonist paricalcitol. ATF6 is known to activate the transcription of several chaperones proteins, including Grp78 [67]. Recent studies also recognize ATF6 as a critical determinant of CHOP dynamics during UPR and ER stress [68]. Taken together, these findings suggest that the inhibitory effects of VDR activation on ER stress may be partly due to the suppression of the STAT3/ATF6 pathway in fibroblasts.

## 5. Conclusions

**Figure 12:**
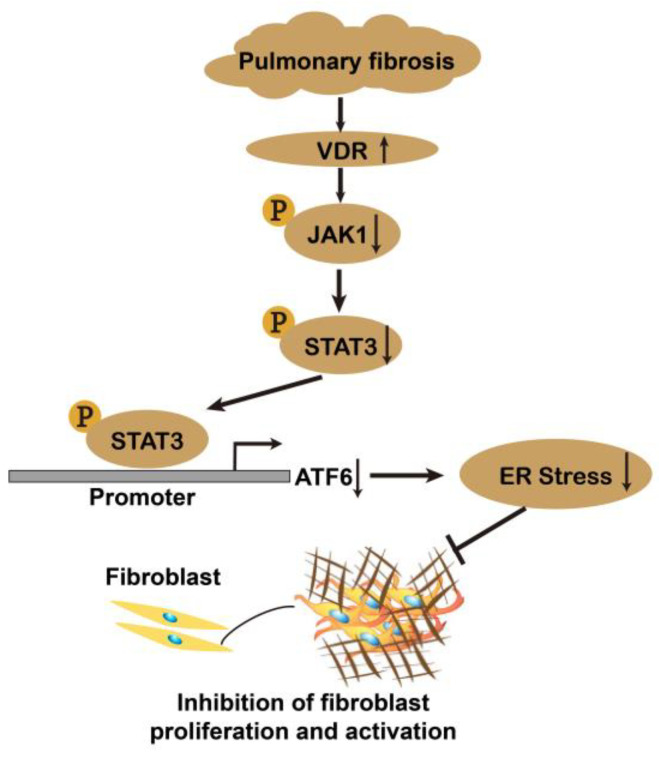
Schematic diagram of the mechanism by which fibroblast upregulation of VDR suppresses lung fibroblast proliferation and activation during pulmonary fibrosis. VDR is specifically upregulated in lung fibroblasts during pulmonary fibrosis. Upon activation, VDR attenuates lung fibroblast proliferation and activation. The anti-fibrotic effects of vitamin D/VDR signaling may be at least partly due to suppression of ER stress in lung fibroblasts. In this study, we have identified JAK1/STAT3 as an upstream regulator of ER stress in fibroblasts. VDR activation inhibits enrichment of STAT3 to ATF6 promoter, thus suppressing ATF6 expression in fibroblasts. Collectively, this study suggests that fibroblast VDR upregulation may be a self-protective response to limit fibroblast proliferation and activation during pulmonary fibrosis by suppressing JAK1/STAT3/ER stress pathway.

The present study revealed, for the first time, that VDR was specifically upregulated in lung fibroblasts during pulmonary fibrosis. The stable knockdown of VDR promoted lung fibroblast proliferation and activation by stimulating the JAK1/STAT3/ER stress pathway. Upon activation, VDR attenuated bleomycin-induced lung fibroblast proliferation and activation in vitro and in vivo. These findings identify JAK1/STAT3 as an upstream regulator of ER stress in fibroblasts. The anti-fibrotic effects of vitamin D/VDR signaling may be at least partly due to the suppression of the JAK1/STAT3/ER stress pathway in fibroblasts (Figure 12). Fibroblast VDR upregulation may be recognized as part of a novel self-protective response to limit fibroblast proliferation and activation during pulmonary fibrosis. 

## Data Availability

The data used to support the findings of this study are available from the corresponding authors upon request.

## Supplementary Materials

The following supporting information can be downloaded at: https://www.mdpi.com/article/10.3390/antiox12081634/s1, Figure S1: Analysis of scRNAseq data of human lung cells shows expression levels of lineage marker genes on UMAP plots of all cells; Figure S2: The effects of VDR agonist on the proliferation of Mlg and MLE-12 cells in the presence of bleomycin; Figure S3: GSEA gene sets associated with well-known fibrosis-associated pathways are enriched in fibroblasts obtained from fibrotic lungs; Figure S4: GSEA gene sets associated with ER stress/UPR-associated pathways are enriched in fibroblasts obtained from fibrotic lungs; Figure S5: Intrapulmonary VDR overexpression suppresses bleo-mycin-induced Grp78 expression in lung fibroblasts in vivo; Table S1: Primer sequences used in the real-time quantitative PCR; Table S2: 8 datasets of lung tissue from healthy control and IPF; Table S3: 12 datasets of lung tissue from healthy control and BLM-induced mouse.
