# Supplementary material for: Fibroblast Upregulation of Vitamin D Receptor Represents a Self-Protective Response to Limit Fibroblast Proliferation and Activation during Pulmonary Fibrosis
Source: Antioxidants (Basel). 2023 Aug 18;12(8):1634. doi: 10.3390/antiox12081634 (PMC10451996; doi:10.3390/antiox12081634)
Supplement: Supplementary file 1 [file antioxidants-12-01634-s001.zip › Supplemental Result.pdf]

Fibroblast upregulation of vitamin D receptor represents a self-protective response to limit fibroblast proliferation and activation during pulmonary fibrosis

Juan Wei[1,3†], Juanhui Zhan[1†], Hui Ji[1], Yitong Xu[1], Qingfeng Xu[1], Xiaoyan Zhu[2*], and Yujian Liu[1*]

Table S1 Primer sequences used in the real-time quantitative PCR

Gene names	the gene accession number	Forward (5′-3′)	Reverse (5′-3′)
VDR	NM_009504.4	CTCCTCGATGCCCACCACAAGACCTACG	GTGGGGCAGCATGGAGAGCGGAGACAG
Ki67	NM_001081117.2	ACCGTGGAGTAGTTTATCTGGG	TGTTTCCAGTCCGCTTACTTCT
Ccne1	NM_007633.2	CTTATGGTGTCCTCGCTGCTTCTG	GCTCGCTGCTCTGCTTCTTACTG
Ccnb1	NM_172301.3	CTGACCCAAACCTCTGTAGTG	CCTGTATTAGCCAGTCAATGAGG
PCNA	NM_011045.2	TTTGAGGCACGCCTGATCC	GGAGACGTGAGACGAGTCCAT
Cdc25b	NM_023117.4	GACAGCTGGAGGAAAACTGA	ATCACTCTCCAGGATGTCCA
Fibronectin	NM_010233.2	CAACAACCGGAATTACACC	GGTCTCGGAGCTGGGAGTAG
Collagen-1	NM_007742.4	GACATGTTCAGCTTTGTGGACCTC	GGGACCCTTAGGCCATTGTGTA
α-SMA	NM_007392.3	CTCCTCAGGACGACAATCGACA	CCTTTCCACAGGGCTTTGTTTG
β-actin	NM_007393.5	TGGAATCCTGTGGCATCCATGAAAC	TAAAACGCAGCTCAGTAACAGTCCG

Table S2 8 datasets of lung tissue from healthy control and IPF

GEO dataset	Platform	Group（n）	Gene. symbol	ID	∣logFC∣	P.Value	References
GSE21369	GPL570 [HG-U133_Plus_2] Affymetrix Human Genome U133 Plus 2.0 Array	Normal (6) VS UIP/IPF (11)	VDR	204254_s_at	0.249	0.487	BMC Med Genomics 2011 Jan; 4: 8.PMID: 21241464
GSE53845	GPL6480 Agilent-014850 Whole Human Genome Microarray 4x44K G4112F (Probe Name version)	Normal (8) VS IPF (40)	VDR	A_23_P162589	0.327	0.091	Thorax 2015 Jan; 70(1): 48-56.PMID: 25217476
GSE38958	GPL5175 [HuEx-1_0-st] Affymetrix Human Exon 1.0 ST Array [transcript (gene) version]	Normal (45) VS IPF (70)	VDR	3452818	0.103	0.101	Thorax 2015 Dec; 70(12): 1138-48. PMID: 26286721, Am J Respir Crit Care Med 2014 Jun; 189(11): 1402-15.PMID: 24779708
GSE110147	GPL6244 [HuGene-1_0-st] Affymetrix Human Gene 1.0 ST Array [transcript (gene) version]	Normal (11) VS IPF (20)	VDR	7962689	0.391	<0.001	Respir Res 2018 Aug; 19(1): 153. PMID: 30111332
GSE2052	GPL1739 Amersham Biosciences CodeLink Uniset Human I Bioarray	Normal (11) VS IPF (13)	VDR	50,28	0.131	0.479	J Exp Med 2006 Dec; 203(13): 2895-906. PMID: 17178917, PLoS Med 2005 Sep; 2(9): e251. PMID: 16128620, Am J Pathol 2008 Mar; 172(3): 583-91.PMID: 18245812
GSE101286	GPL6947 Illumina HumanHT-12 V3.0 expression beadchip	Normal (3) VS IPF (7)	VDR	ILMN_1666203	0.263	0.784	BMC Med Genet 2017 Aug; 18(1): 88.PMID: 28821283
GSE72073	GPL17586 [HTA-2_0] Affymetrix Human Transcriptome Array 2.0 [transcript (gene) version]	Normal (3) VS IPF (5)	VDR	TC12001439.hg.1	0.070	0.567	Respir Res 2015 Oct; 16: 124. PMID: 26453058

GSE48149	GPL16221<br>Illumina HumanRef-8 v3.0 expression beadchip (Search Key version)	Normal (9)<br>VS IPF (13)	VDR	ILMN_8252	0.170	0.196	Arthritis Rheum 2011 Mar; 63(3): 783-94.PMID: 21360508,<br>Front Immunol 2020; 11: 383.<br>PMID: 32210969

Table S3 12 datasets of lung tissue from healthy control and BLM-induced mouse

GEO dataset	Platform	Group(n)	Gene. symbol	ID	∣logFC∣	P.Value	References
GSE103511	GPL6887<br>Illumina MouseWG-6 v2.0 expression beadchip	Control (6) VS BLM (6) 2 weeks	VDR	ILMN_2864062	0.135	0.816	Sci Rep 2018 Jan; 8(1): 1927.<br>PMID: 29386571
GSE94522	GPL10787<br>Agilent-028005 SurePrint G3 Mouse GE 8x60K Microarray (Probe Name version)	Control (3) VS BLM (3) 2 weeks	VDR	A_52_P334562	0.143	0.507	
GSE77326	GPL15887<br>Nimble Gen Mouse gene expression array [100718_MM9_EXP] (gene-level version)	Control (6) VS BLM (6) 3 weeks	VDR	BC006716	0.102	0.645	

GSE43695	GPL6246 [MoGene-1_0-st] Affymetrix Mouse Gene 1.0 ST Array [transcript (gene) version]	Control (3) VS BLM (3) 5 days	VDR	10432032	0.154	0.334	BMC Genomics 2013 Jun; 14:381. PMID: 23758685
GSE25640	GPL1261 [Mouse430_2] Affymetrix Mouse Genome 430 2.0 Array	Control (3) VS BLM (3) 21 days	VDR	1418175_at	0.245	0.104	J Immunol 2011 Jul; 187(1): 450-61. PMID: 21602491
GSE16846	GPL339 [MOE430A] Affymetrix Mouse Expression 430A Array	Control (3) VS BLM (3) 14 days	VDR	1418175_at	0.154	0.244	J Clin Invest 2009 Sep; 119(9): 2550-63. PMID: 19652365
GSE37635	GPL6885 Illumina Mouse Ref-8 v2.0 expression beadchip	Control (7) VS BLM (6) 1 week	VDR	ILMN_2445166	0.26	0.308	Matrix Biol 2013 Oct-Nov; 32(7-8): 424-31. PMID: 23648810
		Control (7) VS BLM (6) 2 weeks		ILMN_2445165	0.195	0.423	
		Control (7) VS BLM (6) 3 weeks		ILMN_2748317	0.042	0.846	
		Control (7) VS BLM (6) 4 weeks		ILMN_2445165	0.515	0.101	
		Control (7) VS BLM (6) 5 weeks		ILMN_2445166	0.407	0.094	
GSE40151	GPL1261 [Mouse430_2] Affymetrix Mouse Genome 430 2.0 Array	Control (8) VS BLM (8) 14 days	VDR	1418176_at	0.053	0.337	PLoS One 2013; 8(4): e59348. PMID: 23565148
		Control (8) VS BLM (8) 21 days			0.071	0.488	
		Control (8) VS BLM (8) 28 days			0.017	0.774	
		Control (8) VS BLM (8) 35 days			0.107	0.071	
GSE42301	GPL2894 GE Healthcare/Amersham Biosciences CodeLink™ UniSet Mouse 20K I Bioarray	Control (3) VS BLM (3) 7 days	VDR	30068	0.062	0.867	Am J Physiol Lung Cell Mol Physiol 2013 May; 304(9): L593-601. PMID: 23457188
		Control (3) VS BLM (3) 1 months			0.302	0.439	
		Control (3) VS BLM (3) 2 months			0.292	0.435	
		Control (3) VS BLM (3) 3 months			0.250	0.465	
		Control (3) VS BLM (3) 4 months			0.045	0.922	
GSE97825	GPL13912 Agilent-028005 SurePrint G3 Mouse GE 8x60K Microarray (Feature Number version)	Control (3) VS BLM (5) 4 days	VDR	61450	0.145	0.117	Am J Respir Crit Care Med 2019 Jul; 200(2): 184-198. PMID: 30964696

GSE112827	GPL13912<br>Agilent-028005 SurePrint G3 Mouse GE 8x60K Microarray (Feature Number version)	Control (3) VS BLM (5) 4 days	VDR	61450	0.157	0.111	Am J Respir Crit Care Med 2019 Jul; 200(2): 184-198.<br>PMID: 30964696
GSE180750	GPL16570<br>[MoGene-2_0-st] Affymetrix Mouse Gene 2.0 ST Array [transcript (gene) version]	Control (3) VS BLM (3)	VDR	17321078	0.112	0.525	

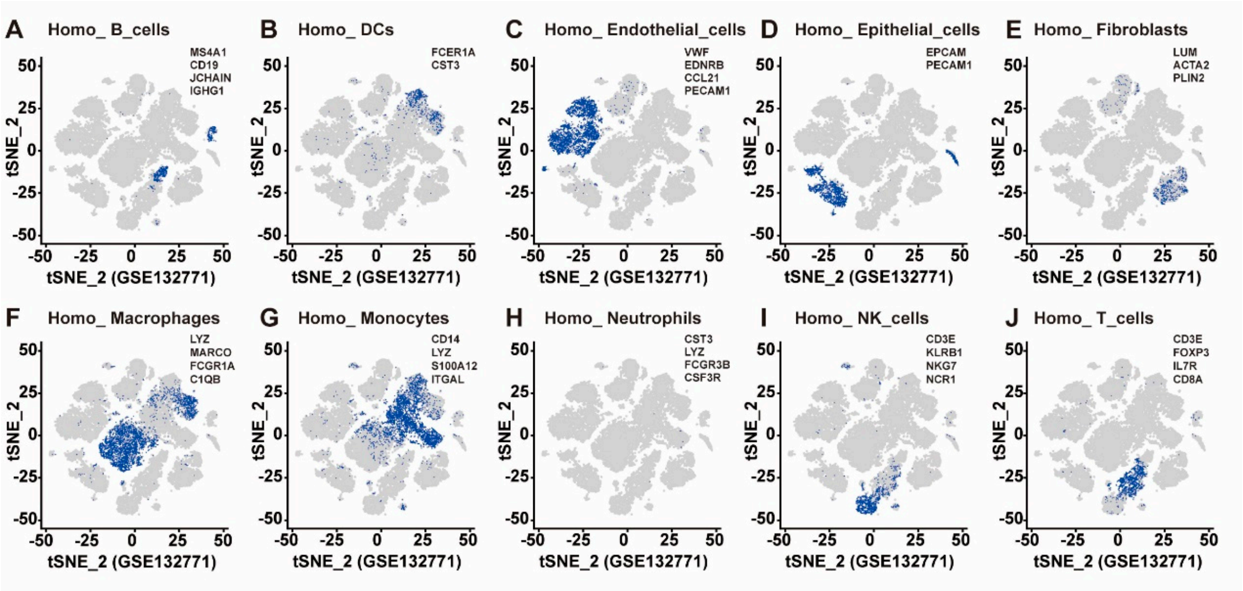

Supplemental Fig. 1 Analysis of scRNAseq data of human lung cells shows expression levels of lineage marker genes on UMAP plots of all cells. Visualization of the lung cell clusters of a published scRNAseq data (GSE132771) was performed on a 2D map with uniform manifold approximation and projection (UMAP). Expression levels of lineage marker genes of B cells (A), dendritic cells (B), endothelial cells (C), epithelial cells (D), fibroblasts (E), macrophages (F), monocytes (G), neutrophils (H), NK cells (I), and T cells (J) were shown on UMAP plots.

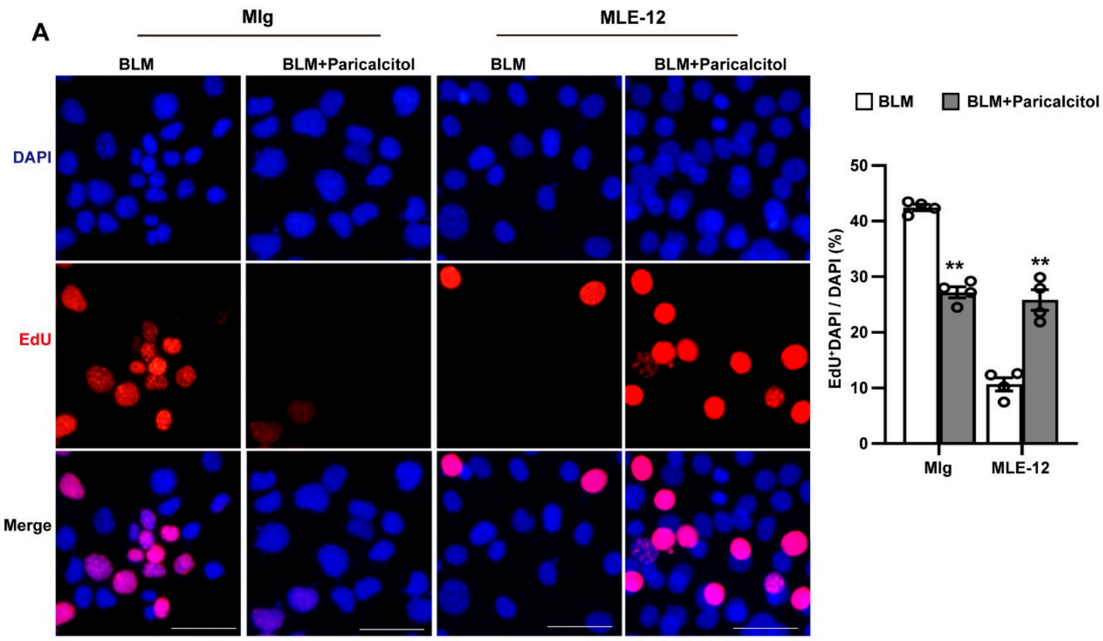

Supplemental Fig. 2 The effects of VDR agonist on the proliferation of Mlg and MLE-12 cells in the presence of bleomycin. Mlg and MLE-12 cells were treated with bleomycin (5 μg/ml) in the presence of vehicle or paricalcitol (2.5 μM) for 48 hours. EdU incorporation assay was used to assess cell proliferation. The proliferative cell number (%) is shown on the right of the representative images as a ratio of the number of EdU+ cells to the total cell number. Data are expressed as means ± SEM (n=4). ** $p < 0.01$ vs BLM. BLM represents bleomycin.

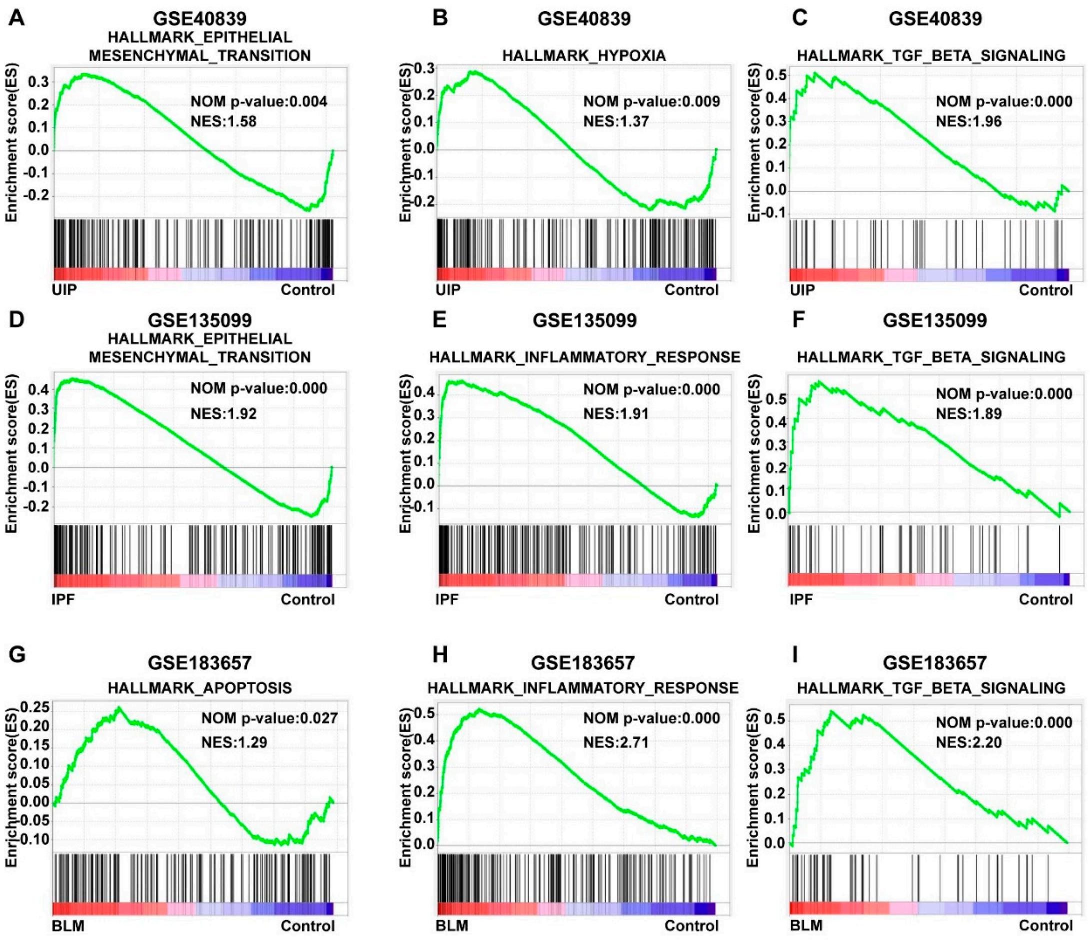

Supplemental Fig. 3 GSEA gene sets associated with well-known fibrosis-associated pathways are enriched in fibroblasts obtained from fibrotic lungs. A-C, GSEA analysis showed that gene sets associated with epithelial mesenchymal transition (A), hypoxia (B), and the TGF-β signaling (C) were significantly enriched in fibroblasts isolated from patients with UIP (GSE40839). D-F, GSEA analysis showed that gene sets associated with epithelial mesenchymal transition (D), inflammatory response (E), and the TGF-β signaling (F) were significantly enriched in fibroblasts isolated from patients with IPF (GSE135099). G-I, GSEA analysis showed that gene sets associated with apoptosis (G), inflammatory response (H), and the TGF-β signaling (I) were significantly enriched in fibroblasts isolated from bleomycin-treated mice (GSE183657).

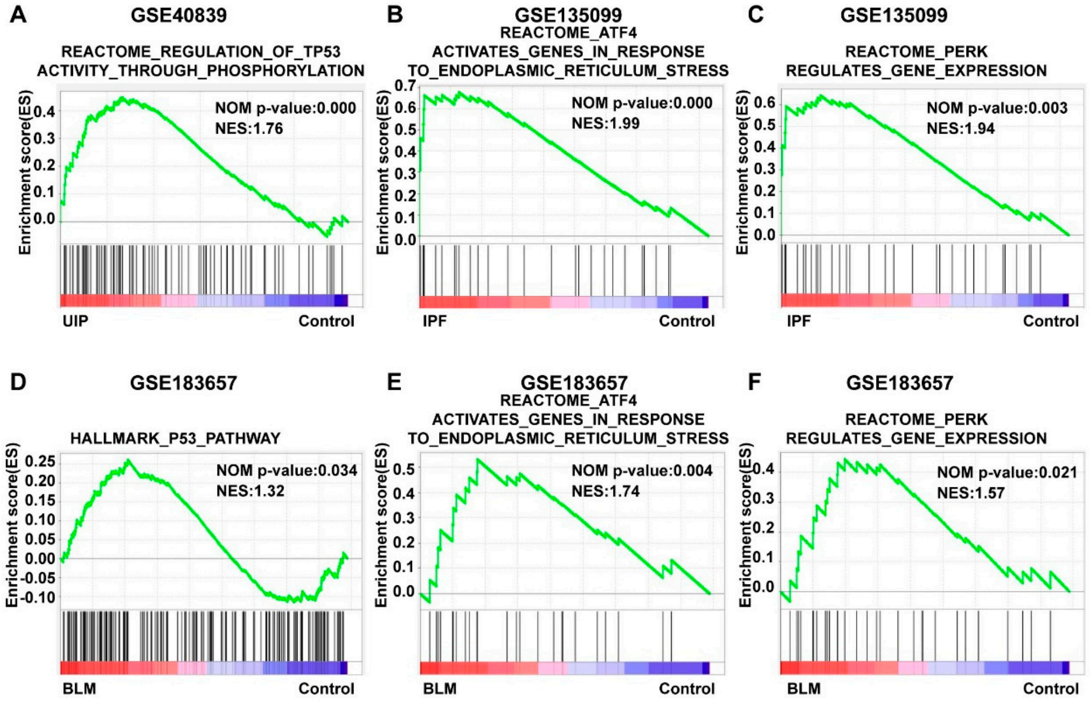

Supplemental Fig. 4 GSEA gene sets associated with ER stress/UPR-associated pathways are enriched in fibroblasts obtained from fibrotic lungs. A, GSEA analysis showed that gene set associated with regulation of TP53 activity through phosphorylation (A) was significantly enriched in fibroblasts isolated from patients with UIP (GSE40839). B-C, GSEA analysis showed that gene sets associated with ATF4 activates genes in response to endoplasmic reticulum stress (B) and PERK regulates gene expression (C) were significantly enriched in fibroblasts isolated from patients with IPF (GSE135099). D-F, GSEA analysis showed that gene sets associated with p53 pathway (D), ATF4 activates genes in response to endoplasmic reticulum stress (E), and PERK regulates gene expression (F) were significantly enriched in fibroblasts isolated from bleomycin-treated mice (GSE183657).

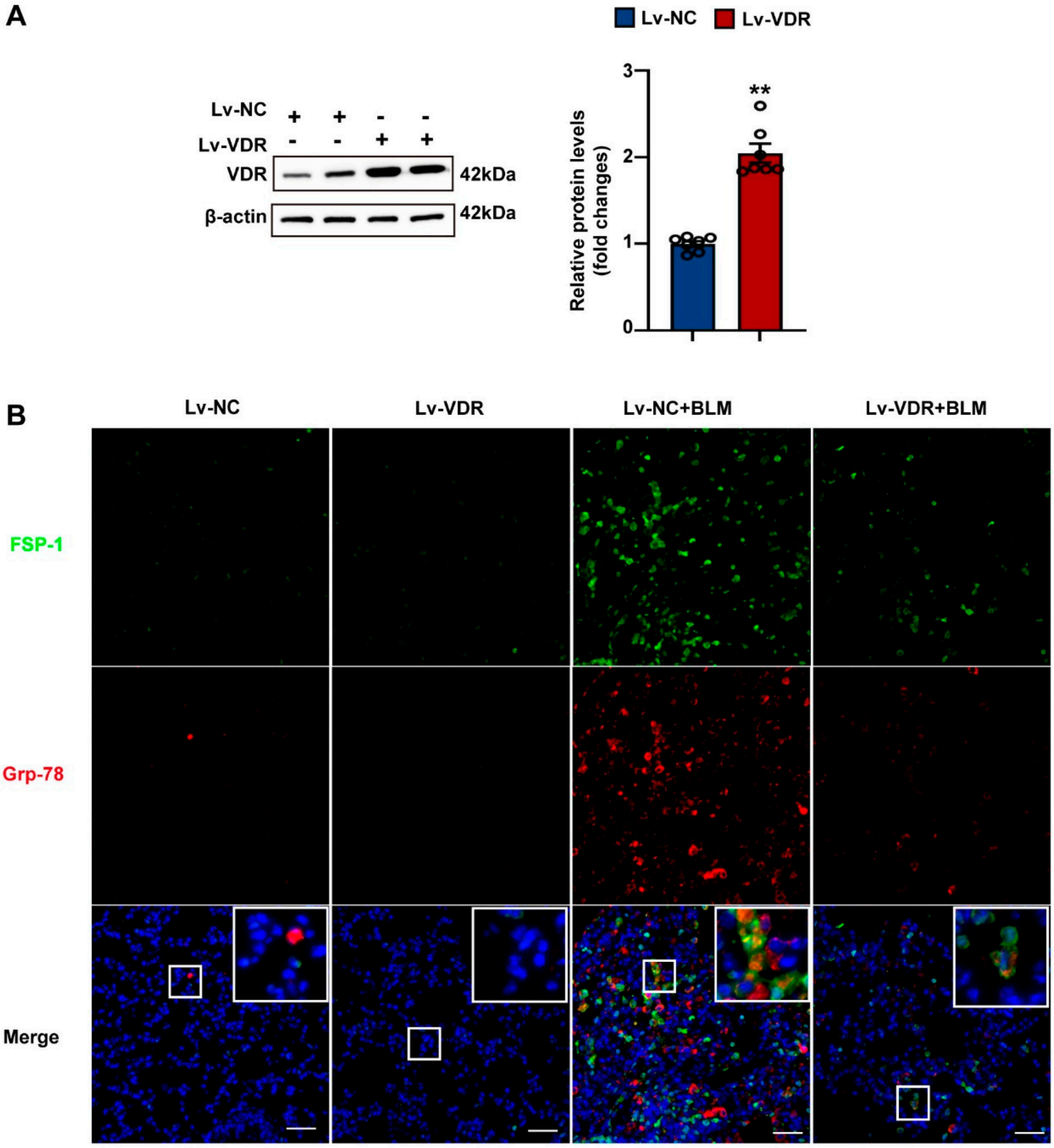

Supplemental Fig. 5 Intrapulmonary VDR overexpression suppresses bleomycin-induced Grp78 expression in lung fibroblasts in vivo. A, Mice were intratracheally instilled with Lv-VDR or Lv-NC. Two weeks later, lung tissues were harvested to determine VDR expression by western blot analysis. Representative protein bands were presented on the left of the histograms. B, Mice were randomly divided into four groups: Lv-NCl, Lv-VDR, Lv-Control+BLM, and Lv-VDR+BLM. Lung sections were stained with fluorophore-labeled antibodies against Grp78 (Alexa Fluor 647, red) and fibroblast marker FSP-1 (Alexa Fluor 488, green). DAPI staining was used to detect nuclei (blue). Merge image represented double positive staining for FSP-1 and Grp78. Areas in white boxes were shown enlarged. Scale bars correspond to 50 μm. BLM represents bleomycin.
